# Beneficial Health Effects of Glucosinolates-Derived Isothiocyanates on Cardiovascular and Neurodegenerative Diseases

**DOI:** 10.3390/molecules27030624

**Published:** 2022-01-19

**Authors:** Ramla Muhammad Kamal, Ahmad Faizal Abdull Razis, Nurul Syafuhah Mohd Sukri, Enoch Kumar Perimal, Hafandi Ahmad, Rollin Patrick, Florence Djedaini-Pilard, Emanuela Mazzon, Sébastien Rigaud

**Affiliations:** 1 Natural Medicines and Products Research Laboratory, Institute of Bioscience, Universiti Putra Malaysia, Serdang 43400, Selangor, Malaysia; amlaamal1990@gmail.com; 2 Department of Pharmacology, Federal University Dutse, Dutse 720101, Jigawa State, Nigeria; 3 Department of Food Science, Faculty of Food Science and Technology, Universiti Putra Malaysia, Serdang 43400, Selangor, Malaysia; 4 Faculty of Applied Science and Technology, Universiti Tun Hussein Onn Malaysia, Batu Pahat 86400, Johor, Malaysia; aw170150@siswa.uthm.edu.my; 5 Department of Biomedical Sciences, Faculty of Medicine and Health Sciences, Universiti Putra Malaysia, Serdang 43400, Selangor, Malaysia; enoch@upm.edu.my; 6 Department of Veterinary Preclinical Sciences, Faculty of Veterinary Medicine, Universiti Putra Malaysia, Serdang 43400, Selangor, Malaysia; hafandi@upm.edu.my; 7 Université d’Orléans et CNRS, ICOA, UMR 7311, BP 6759, CEDEX 02, F-45067 Orléans, France; patrick.rollin@univ-orleans.fr; 8 LG2A UMR 7378, Université de Picardie Jules Verne, 33 rue Saint Leu—UFR des Sciences, F-80000 Amiens, France; florence.pilard@u-picardie.fr (F.D.-P.); sebastien.rigaud@u-picardie.fr (S.R.); 9 Laboratorio di Neurologia Sperimentale, IRCCS Centro Neurolesi "Bonino Pulejo", 98124 Messina, Italy; emazzon.irccs@gmail.com

**Keywords:** cardiovascular diseases, neurodegenerative diseases, Cruciferae vegetables, phytochemicals, glucosinolates, isothiocyanates

## Abstract

Neurodegenerative diseases (NDDs) and cardiovascular diseases (CVDs) are illnesses that affect the nervous system and heart, all of which are vital to the human body. To maintain health of the human body, vegetable diets serve as a preventive approach and particularly Brassica vegetables have been associated with lower risks of chronic diseases, especially NDDs and CVDs. Interestingly, glucosinolates (GLs) and isothiocyanates (ITCs) are phytochemicals that are mostly found in the Cruciferae family and they have been largely documented as antioxidants contributing to both cardio- and neuroprotective effects. The hydrolytic breakdown of GLs into ITCs such as sulforaphane (SFN), phenylethyl ITC (PEITC), moringin (MG), erucin (ER), and allyl ITC (AITC) has been recognized to exert significant effects with regards to cardio- and neuroprotection. From past in vivo and/or in vitro studies, those phytochemicals have displayed the ability to mitigate the adverse effects of reactive oxidation species (ROS), inflammation, and apoptosis, which are the primary causes of CVDs and NDDs. This review focuses on the protective effects of those GL-derived ITCs, featuring their beneficial effects and the mechanisms behind those effects in CVDs and NDDs.

## 1. Introduction

Numerous diseases have resulted in people receiving poor quality healthcare as a consequence of aging-related affections, mutations, generational disorders, cancers, or low body metabolism, particularly in elderly people who wish to remain active and productive in their lives. The diseases that are most commonly encountered in the elderly are related to both nervous and heart disorders, which have raised the mortality rate in the elderly population, but also affect the younger generation [1]. Neurodegenerative diseases (NDDs) and cardiovascular diseases (CVDs) are illnesses that affect prime important systems in the human body; nervous and cardiovascular systems, respectively [2,3,4,5,6]. Some studies have been published on the use of phytochemicals’ chemopreventive effects as therapies to help reduce the impact of these diseases [7,8]. They are also used to treat infectious diseases as well as to encourage overall health [9,10].

A wide variety of plant-based compounds, often known as phytochemicals, have been identified as having promising chemopreventive activity [11]. Plants have developed phytochemicals as a secondary defense mechanism, and they possess biological elements that are beneficial to human health [9]. Glucosinolates (GLs) and isothiocyanates (ITCs) are phytochemicals that have been found to protect the heart and brain [10]. These metabolites are commonly found in the Cruciferae family, which differentiates it from other plants [9]. Plant antioxidants and anti-inflammatories are increasingly being used to defend against a wide range of cardiovascular and neurological illnesses [7,12,13,14]. Bioactive substances produced from natural sources have grown in importance in modern medicine as they lessen the risk of CVDs and NDDs by scavenging free radicals and or averting their generation [7,8,9].

Cabbage, broccoli, cauliflower, kale, brussels sprouts, kohlrabi, rape, black and brown mustard, daikon, wasabi, turnips, and rutabagas are all cruciferous vegetables containing various GLs and ITCs [15,16] that have been shown to alleviate CVDs and NDDs [17] and also prevent cancer growth and metastasis [18]. GLs and ITCs are also present in the tropical plant *Moringa oleifera* Lam. (Moringaceae), showing similar protective effects [19]. The pungent flavor of those vegetables, and condiments and flavoring agents prepared from them, is due to ITCs resulting from the myrosinase reaction, which occurs quickly when plant tissue is damaged [20,21]. The extreme degree of metabolic specialization, along with the deterioration of cellular organelles, which occurs during the differentiation of glucosinolate-sulfur-rich cells (S cells) highlights the significance of this reaction for plant defense and generation of bioactive compounds beneficial to man [16].

## 2. Origin and Synthesis of Glucosinolates (GLs) and Isothiocyanates (ITCs)

Glucosinolates are primarily phytochemicals found in the Cruciferae (Brassicacea) family of plants; first discovered in *Brassica oleracea, Brassica nigra, Brassica hirta, Brasssica campestris,* and *Brassica napus* [20]. However, sporadic occurrence in other plants has been reported like in the seed and latex of papaya (*Carica papaya* L.) [20] and recently in the seeds and leaves of *Moringa oleifera* Lam. plant [22,23]. Plants utilize GLs as protection from stressful conditions arising from environmental changes, pathogens, and so on [15,24]. Differences in precursor amino acids give rise to different GLs classified as; aliphatic GLs, derived from methionine, isoleucine, leucine, or valine; aromatic GLs, derived from phenylalanine or tyrosine; and indole GLs, derived from tryptophan [25]. GLs are synthesized from these amino acids through a series of reactions involving *N*-hydroxylation, oxidative decarboxylation, and oxidation leading to the formation of a nitro group to which cysteine incorporation produces thiohydroxymic acid [26]. Glucose molecule transfer to thiohydroxymic acid from UDP-glucose leads to the formation of desulphoglucosinolate and finally GL is formed by sulphate addition from the donor 3’-phosphoadenosine-5’-phosphosulfate (PAPS) [20].

Upon harm to plant tissue by cutting for example or during chewing, GL comes into contact with an enzyme myrosinase (thioglucoside glucohydrolase, EC 3:2:3:1) as under normal conditions they are physically separated [27]. This heralds myrosinase catalyzed hydrolysis of GLs with generation of unstable aglucone that rapidly rearranges to ITCs and other aglucone; nitriles, thiocyanates, oxazolidine-2-thiones, hydroxynitriles and epithionitriles depending on the structure of the parent GL, genotype, plant or pests specifier proteins (SPs) and the presence of factors which modify the action of myrosinase such as temperature, pH, drying method, duration and temperature of plant storage, high dilution with water, as well as hydrolysis with exogenous myrosinase [24,27]. ITCs thus generated, undergo spontaneous cyclization to the corresponding oxazolidine-2-thione [20].

## 3. Glucosinolates (GLs)

Naturally produced by plants, glucosinolates (GLs) are sulphur-containing secondary metabolites, which are widely found in plants of the order Brassicales, and particularly in the Brassicaceae family [28]. Over 130 different side-chains have been reported for GLs in the literature [29]. There have been considerable advances in scientific knowledge on how GLs and their derivatives potentially serve to reduce the incidence of heart and neurological disease when taken as part of a healthy diet [30]. Moreover, researchers have shown much interest in identifying how consuming diets rich in these natural antioxidants confers protection against CVDs and NDDs [17].

**Figure 1:**
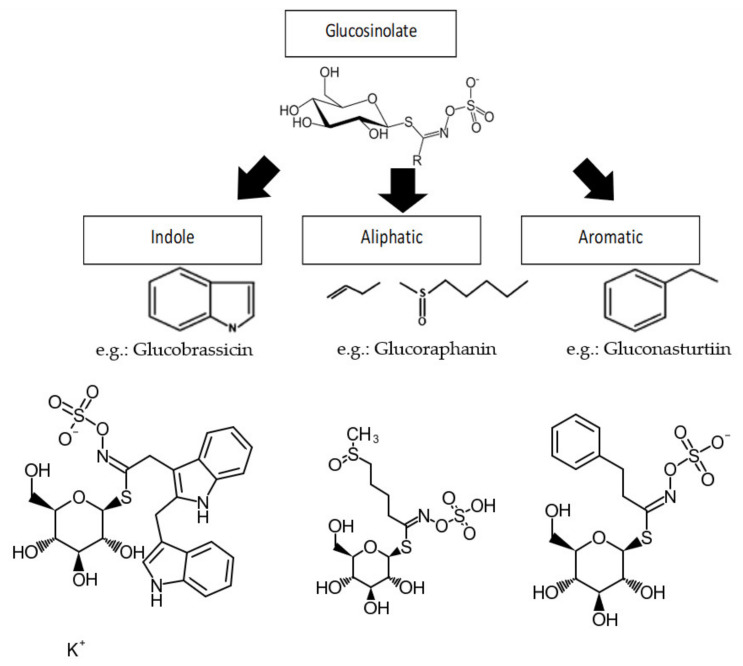
Three major classes of amino acid precursors of GLs side chains (R). Structures were retrieved from PubChem [32,33,34].

All known GLs display a striking structural homogeneity based on a β-D-glucopyrano unit, an *O*-sulfated anomeric *(Z)*-thiohydroximate function connected to a hydrophobic side chain R which constitution, depending on plant species, is the sole structural variant. There are three major types of side chains based on amino acids precursors: aliphatic GLs (e.g., glucoraphanin), aromatic GLs (e.g., gluconasturtiin), and indole GLs (e.g., glucobrassicin) (Figure 1) [25,31]. However, merely five of these GLs, glucobrassicin, sinigrin, glucoraphasatin (dehydroerucin), glucoraphanin, and glucoiberin, were associated with human diet, according to the European Prospective Investigation into Cancer and Nutrition (EPIC) study [31]. Though, another important GL has been discovered in the edible *M. oleifera* Lam. plant with very similar structural definition [22].

**Figure 2:**
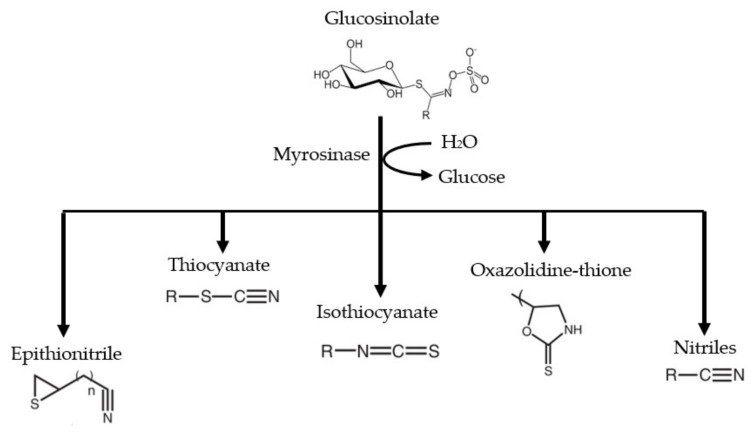
Selected products after hydrolysis by myrosinase enzyme. The compounds produced depend on side chain (R) and other factors during the conversion process. Adapted from Fuentes et al. [35].

GLs are physiologically inactive chemicals, but once they are hydrolyzed by myrosinase enzyme, they are converted into biologically active compounds such as ITCs, thiocyanates, nitriles, epithionitriles, oxazolidinethiones, and so forth (Figure 2) [15]. The differences in GL side chains resulted in a broad variety of ITCs through enzymatic hydrolysis [35]. For example, glucoraphanin (GRA) produces sulforaphane (SFN), sinigrin (SIN) yields allyl isothiocyanate (AITC), gluconasturtiin (GST) gives rise to phenethyl isothiocyanate (PEITC), glucoerucin (GER) to erucin (ER), glucotropaeolin (GTL) produces benzyl isothiocyanate (BITC), while glucomoringin (GMG) yields moringin (MG) [36,37]. It is well documented that the breakdown products of GLs have beneficial effects, and much research has unveiled protective effects of these compounds against CVDs, neurodegeneration, diabetes, and several other inflammatory disorders [25].

## 4. Isothiocyanates (ITCs)

Isothiocyanates R-N=C=S are a class of molecules in which R is an alkyl or aryl group: these compounds can be formed through hydrolysis of the GLs present in ingested cruciferous vegetables. ITCs are highly vulnerable to nucleophilic attacks at the electron-deficient functional carbon atom, often referred to as reactive site [38]. Numerous studies have also referenced ITCs as antioxidants, notably in relation to phase II enzyme stimulation, while just a few have demonstrated direct antioxidant activity [39]. They also exhibit anti-inflammatory, antimicrobial, neuroprotective, and cardioprotective properties among others [10,40]. ITCs are generally regarded as safe, with no major negative effects observed in humans [10,16].

**Figure 3:**
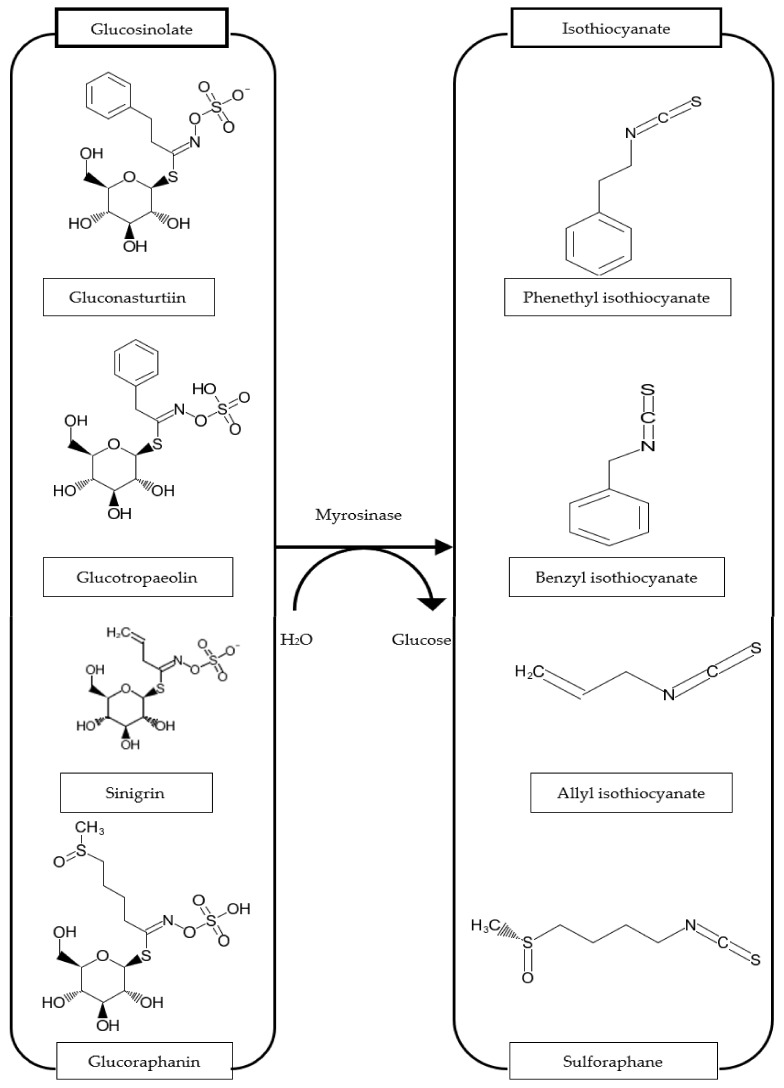
Isothiocyanates issued from their glucosinolate precursors. Structures were retrieved from PubChem [33,34,52,53,54,55,56,57].

The structures of ITCs plainly arise from the side chain of their GL precursors as depicted above (Figure 3). The actions of cutting or chewing the vegetables enable activation of the enzyme myrosinase, while heating reduces its activity. Still, after consumption of cruciferous vegetables, the microbial myrosinase from the intestines can release ITCs in the digestive tract [41]. This type of hydrolysis reaction has been detected in the bacteria of the human gut [42,43,44] and in mice [45]. According to existing literature, myrosinase and ITCs are thermolabile [46], thus, consuming veggies in the raw form releases higher ITCs, than after being processed by heating [47]. ITCs are conjugated with glutathione to dithiocarbamates by glutathione S-transferases (GST). Dithiocarbamates are cleaved via mercapturic acid pathway to cysteine-ITC conjugates, then become converted to the corresponding *N*-acetyl-S-cysteine conjugates by *N*-acetyl transferase [10,30,48,49,50,51].

## 5. Mechanism of Cardio- and Neuroprotective Effect

An accumulation of reactive oxygen species (ROS) in cells causes oxidative stress, which is responsible for a wide range of pathophysiological disorders, including CVDs and NDDs [58]. For instance, excessive ROS production causes nitric oxide (NO) depletion and vasoconstriction, which results in arterial hypertension [59]. Due to different anatomic and physiological linkages between the brain and the heart, there are diverse correlations between normal and abnormal behaviors in both systems (brain–heart diseases) [3]. There are several inherited and non-inherited central nervous system (CNS) diseases that can impact the heart in various ways, but comprehensive prospective research is scarce and data on the subject remain anecdotic [3].

Following the breakdown of particular classes of GLs by myrosinase, a variety of ITCs can be produced as highlighted in Section 3. Each of these bioactive components is recognized to be a powerful antioxidant in the fight against CVDs and NDDs [17]. Several ITCs have been recorded in the literature on their performance in the treatment of such crucial disorders (Table 1) [10,17]. ITCs are known to be strong nuclear factor erythroid 2-related factor 2 (Nrf2) activators through the activation of antioxidant response element (ARE)-driven genes, as well as strong suppressors of inflammatory response via the NF-κB (nuclear factor kappa-light-chain-enhancer of activated B cells) pathway [60]. Apoptosis suppression is another important effect responsible for cardio- and neuroprotection by ITCs through arresting the mitochondrial apoptotic pathway [16].

### 5.1. Cardioprotective Effect

Cardiovascular disorders contribute to an unprecedented proportion of global chronic diseases and will continue to be the leading cause of death in the predictable future [2]. Oxidative stress plays a critical role in the pathogenesis of cardiac diseases [119]: increased production of reactive oxygen and/or nitrogen species has been implicated in the development of ischemic myocardial injury and cardiac dysfunction throughout many reports [120]. A multitude of events can produce oxidative stress, which involves generation of ROS and cellular oxidative damage [121]. Cardiovascular remodeling and fibrosis are exacerbated by oxidative stress, which eventually leads to heart failure [63]. Similarly, inflammation contributes to onset and progression of CVDs [122,123,124] and cardiomyocyte apoptosis worsens disease outcome [7]. Throughout documented research, the use of GLs and ITCs has been shown to retard or reduce the effects and phenomena associated with CVDs [10,16,17].

Release of the gasotransmitter hydrogen sulfide (H₂S) from those sulfur compounds is a novel mechanistical facet of the cardioprotective effect of ITCs [125]. This has laid sound ground for researchers to understand the basis for pharmacological and nutraceutical properties of ITCs [126]. ITCs release H₂S by forming adduct with cysteine, then the adduct undergoes intramolecular cyclization with the liberation of either H₂S and dihydrothiazole derivatives or organic amine and raphanusamic acid [127,128,129]. In the study by Testai et al. [130] the H₂S-donor 4-carboxyphenyl isothiocyanate protected against ischemia/reperfusion (I/R)-mediated myocardial tissue injury [130]. The endogenous cystathionine-γ-lyase (CSE)/H₂S system protected arteries from atherosclerosis through modulating cPKCβII/Akt signal pathway in mouse uremia accelerated atherosclerosis (UAAS) model [131]. Future studies may uncover whether ITCs modulate this pathway. Among GLs/ITCs, sulforaphane, glucoraphanin, glucomoringin-isothiocyanate, and sinigrin are those mainly associated with cardioprotective activity [132], but we shall also review other GLs/ITCs based on more recent research. In particular, moringin normalizes blood glucose and cholesterol profile in diabetes mellitus models and stimulates heart function in CVD models [40]. Importantly, Arauna et al. [132] pointed out that ITCs have a therapeutic role in the elderly with CVD [132].

#### 5.1.1. Sulforaphane (SFN)

**Figure 4:**
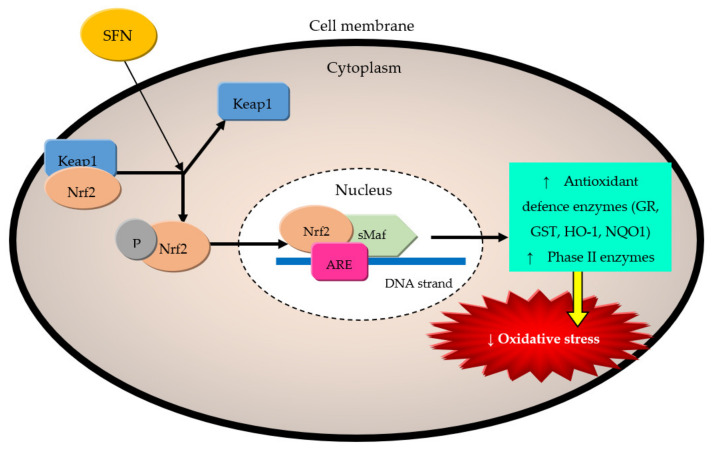
Pathway activation of Nrf2 (nuclear factor erythroid-2 related factor 2) by isothiocyanates (ITCs). Adapted from Angeloni et al. [136].

The revolving causative factors in cardiovascular risk and diseases are as highlighted above; the roles of sulforaphane (SFN) in alleviating oxidative stress [36,133], inflammation [61,134], and apoptosis [135] have been well documented. The ability of SFN to modulate Nrf2/Keap (Nrf2/kelch-like ECH-associated protein 1), NF-κB, AGE/RAGE (advanced glycation end products/receptor for advanced glycation end-product), and adenosine 5‘-monophosphate (AMP)-activated protein kinase (AMPK) pathways as well as adhesion molecules expression have been correlated with protection against cardiovascular related inflammation, atherosclerosis, hypertension (HT), diabetes mellitus (DM), cardiomyopathy and heart failure (HF) [126]. SFN suppressed lipid peroxidation and lowered total ROS levels by 40% in adult cardiomyocytes through Nrf2- and PGC-1α (peroxisome proliferator-activated receptor co-activator-1 alpha)-mediated increased expression and activity of superoxide dismutase (SOD), both SOD-1 (cytosolic) and SOD-2 (mitochondrial) isoforms [133]. Glutathione is the most prevalent endogenous antioxidants utilized to prevent cells from oxidative damage on a daily basis, and through activation of the Nrf2/ARE pathway, SFN significantly raised the concentration of active glutathione [136]. As a typical Nrf2 activator, SFN modified Keap1 (Keap1 maintains Nrf2 in a dormant state under physiological conditions) conformation by associating with the thiol groups of particular cysteine residues within Keap1 and prevented Nrf2 from being ubiquitinated and degraded (Figure 4) [136].

Further, it was found that when SFN was given at a concentrated dose of 10 µM, the quantity of Nrf2 increased by 1.4 times [137]. The release of Nrf2 by SFN has been postulated as the reason for SFN’s long-term and significant effects in the prevention of CVDs [137]. By upregulating the Nrf2/ARE pathway, SFN caused increased expression and activity of Nrf2 responsive genes (NAD(P)H: quinone oxidoreductase (NQO-1), heme oxygenase-1 (HO-1), and glutamate cysteine ligase (GCL)), thereby ameliorating pathogenesis in CVDs [18,35,43,44]. On the other hand, SFN modulated the MAPK/AP-1/NF-κB pathway(MAPK-mitogen-activated protein kinase [138,139]; it suppressed activation of p38 [138], ERK (extracellular regulated protein kinases), and JNK (c-jun N-terminal kinase) [140], decreased c-Jun and c-Fos expression [140], and inhibited ΙκΒ kinase (IĸKβ) activation, degradation of ΙκΒ-α (inhibitor of NFĸB) and NF-κB/p65, thereby contributing to amelioration of inflammation associated with CVDs.

SFN was reported to reduce obesity by inhibiting adipogenesis (causes cell cycle arrest) and lipogenesis [141,142]. SFN lowered serum lipids (but increased high-density lipoprotein-cholesterol-HDL-C), leptin, plasma insulin, HOMA-IR (homeostatic model assessment of insulin resistance), glucose, and reduced body weight and BP (both systolic and diastolic) [61,141,143]. In atherosclerosis model (LPS-stimulated Bovine aortic endothelial cells/plasmid transfected endothelial cells/HUVECS/VSMC), SFN reduced the expression of intracellular adhesion molecule (ICAM-1) [144], VCAM-1 (vascular cell adhesion molecule-1) [138,140], E-selectin, and MCP-1 (monocyte chemotactic protein-1) [138,145]. This strongly inhibited binding of monocytes to endothelial cells [138,139,144]. SFN also inhibited the generation of ROS [140] and the damaging effect of oxidized low-density lipoprotein (ox-LDL) on endothelium [139]. SFN inhibition of ICAM-1 expression has been through Rho A/ROCK/NF-κB signaling pathway downregulation, which also contributed to suppression of inflammation within atherosclerotic plaques [146].

Similarly, due to TNF- α-induced vascular inflammation in C57BL/6 mice, SFN reduced serum levels of adhesion molecules, prevented vascular inflammation, and endothelial disruption [145]. SFN suppressed vascular injury in diabetes via inhibiting gene expression of RAGE, MCP-1, ICAM-1, VCAM-1 and binding of THP-1 human monocytic cells to human umbilical vein endothelial cells (HUVECs). SFN also reduced expression of 8-hydroxy-2′-deoxy-guanosine (8-OHdG), a marker of oxidative stress, as well as lymphocyte infiltration and NADPH (nicotinamide adenine dinucleotide phosphate) oxidase activity [61]. Glutathione has been shown as essential in preventing monocyte adhesion to endothelium by SFN as the lower the concentration of intracellular GSH, the higher the suppressive effect due to SFN and vice versa [144]. In addition to the foregoing findings, SFN normalized triglycerides (TG), HDL-C, and diastolic blood pressure in the in vivo model (AGEs-induced inflammation in rat aorta) [61].

Again, SFN inhibited GATA binding factor 6 (GATA6) expression and its binding to VCAM promoter gene as well as proliferation and migration of vascular smooth muscle cells (VSMCs) and hence inhibited restenosis after vascular injury in a rat carotid artery balloon injury model [147]. Inhibition of vascular inflammation and VSMC proliferation and migration by SFN contributed to suppression of neointima formation in obese rats with femoral arterial injury [143], and this effect was later shown to be via suppression of the NOX4/ROS/Nrf2 pathway [148]. In addition to its effect on substantially reducing neointimal development, SFN expanded arterial luminal width and suppressed the expression of proliferating cell nuclear antigen (PCNA) [149]. SFN suppressed both development and progression of atherosclerosis as shown in hypercholesterolemic rabbits: it improved endothelial function (i.e., aortic relaxation and normalized intima/media (I/M) ratio) probably through its antioxidant (normalized aortic nitrate and serum antioxidants), anti-inflammatory (lowered C-reactive protein (CRP) and lactate dehydrogenase (LDH), decreased NF-κB expression), and anti-lipidemic (normalized serum lipids) effects [150]. Mechanistically, chromatin immunoprecipitation (ChIP) and electrophoretic mobility shift assay (EMSA) revealed SFN’s inhibition of endothelial lipase expression by preventing binding of NF-κB to endothelial lipase promoter gene (LIPG) (Lipase G, Endothelial Type), hence preserved HDL, which is beneficial in reducing atherosclerosis [151].

In an experimental model of myocardial infarction (MI), SFN via modulating the MAPK pathway improved morphological, histological, and echocardiographic parameters of MI vis-à-vis decrease of cardiac fibrosis and cardiac dilation [135]. Moreover, SFN reduced autophagy in MI compared to control and disease groups by normalizing the ratio of autophagy-related signaling proteins p-AMPK/AMPK and microtubule-associated protein (MAP) 1 light chain 3 (LC3I/II) [135]. During reperfusion phase, SFN curtailed further myocardial damage and this helped in reducing infarct size more effectively than the conventional mechanical maneuver of post-conditioning (PostC), which made the authors propose that the transient elevation of Nrf2 activation with upregulation of both phase I and II enzymes serves as a long-term cardioprotective mechanism against experimental myocardial ischemia/reperfusion injury in Wistar rats [62]. Cardioprotection by SFN during reperfusion was shown to rely on AhR (aryl hydrocarbon receptor) activation and increased expression levels of Nrf2, NQO-1, and HO-1. Additionally, SFN reduced interleukin (IL)-1 beta (IL-1β), IL-6, and tumor necrosis factor alpha (TNF-α) as well as increased activation of ERK1/2, glycogen synthase kinase 3 beta (GSK-3β) and protein kinase C (PKC) [62]. Moreover, following reperfusion, SFN decreased ROS production and malondialdehyde (MDA) level, increased SOD, matrix metalloproteinases (MMP), and B-cell leukemia/lymphoma-2 (Bcl-2) expression (anti-apoptotic factor) and also reduced Bcl associated X protein (Bax) and caspase-3 expression (pro-apoptotic factors). Appreciable reduction of creatine kinase (CK) and increment of NO were also recorded [152].

With regards to chronic heart failure (CHF) induced with aortic constriction in rabbits, SFN for 12 weeks led to the suppression of atrial natriuretic peptide (ANP) and brain natriuretic peptide (BNP), reduction of collagen I and collagen III expression in heart tissues (which decreases cardiac fibrosis) resulting in improved cardiac function and remodeling [63]. In another study, SFN suppressed cardiac hypertrophy by inhibiting MAPK signaling pathways and GATA4/GATA6 expression with downregulation of ANP, BNP, and beta myosin heavy chain [153]. When compared to both control and disease groups, SFN improved left ventricular systolic and diastolic activity (lowered left ventricular ejection fraction (LVEF), left ventricular shortening fraction (LVFS), left ventricular end-systolic diameter (LVESD), and left ventricular end-diastolic diameter (LVEDD)), but interventricular septal thickness (IVS), left ventricular posterior wall thickness (LVPW), and left ventricular end diastolic pressure (LVEDP) remained unaltered [63]. In doxorubicin-induced CHF, SFN increased Nrf2 gene expression and transcriptional activity (NQO-1, HO-1) as well as inhibited the expression of inflammatory factor plasminogen activator inhibitor-1 (PAI-1) and fibrotic factor, connective tissue growth factor (CTGF) [134,154,155]. Epigenetic modification of histone deacetylases (HDAC) by SFN may serve as a novel mechanism in CHF treatment [156].

In a model for arrhythmia (isoproterenol-induced cardiac stress in rat), SFN-enriched broccoli extract given for two weeks reduced sympathetic drive and increased parasympathetic effect with demonstrable normalization of heart rate and improvement of left ventricular function [64]. SFN reduced pulmonary arterial pressure by abolishing right ventricular and lung inflammation, fibrosis, and the attendant remodeling and dysfunction [65]. This was akin to upregulation of the Nrf2 responsive gene NQO-1 and downregulation of the inflammatory mediator NLRP3 (nucleotide-binding domain leucine-rich repeat-containing (NLR) family pyrin domains-containing protein 3) [65]. Similarly, SFN improved features of cardiomyopathy through epigenetic modification of Nrf2 activation, associated with inhibited HDAC enzyme activity, which contributed to long-lasting cardioprotection [157]. SFN in combination with zinc prevented development of diabetic cardiomyopathy in type I DM OVE 26 (OVE) mice in a synergistic manner. The combo increased Nrf2 activity and metallothionein expression better than either given alone [158]. Aging has been shown to be associated with heart and skeletal muscle dysfunction and glucose intolerance, but these were suppressed by SFN, which reduced 8OHdG (8-hydroxy-2-deoxyguanosine) and muscle myostatin expression—markers of skeletal muscle oxidation and apoptosis respectively—as well as upregulated expression of antioxidant and anti-electrophilic genes (SOD1, SOD2, CAT-catalase, Nrf2, and Akr3- aldo-keto reductase 3) [66]. Overall, SFN improved ejection fraction, fractional shortening, stroke volume, and cardiac output with reduced mortality compared to control group [66]. Based on the fact that chronic hexavalent chromium exposure is an evolving threat to human and animal health, Yang and colleagues [159] went on to discover that SFN can neutralize toxin-induced cardiac oxidative stress, apoptosis, and structural and functional disruption. On one hand, SFN increased expression of Sesn2, which in turn stimulated the AMPK/Nrf2 pathway and on the other inhibited NF-κB and mitochondrial apoptotic pathways [159]. With extension to vascular effects in tumor pathogenesis, SFN potently inhibited vascular endothelial growth factor (VEGF) and its receptor as well as key angiogenic transcription factors and this translated to decreased formation of new microcapillaries [160].

SFN has been reported as safe in humans, and it reduced side effects of co-administered anticancer medications like doxorubicin. SFN has been approved for clinical studies as a dietary supplement against obesity; though some studies have been done already, researchers are yet to achieve their goal of its clinical efficacy [134]. Unfortunately, in a certain population of humans, SFN may produce adversity as it is not without some setbacks. Rhoden et al. [161] found impairment of mitochondrial function and frank sterling effect of cardiomyocyte stretch with negative inotropic effect when SFN was administered as both acute (30 μM) and chronic (1 μM for 3 weeks) dosing in in vitro (EHTs from neonatal (0 to 3 day old) Wistar rats cardiomyocytes (NRCMs) and human-induced pluripotent stem cell-derived cardiomyocytes (hiPSC-CMs) and ex vivo (hearts from adult male C57 Black Swiss mice) heart studies [161]. With the approval of SFN for clinical trials [134], authors warn caution in patients with cardiac-related illnesses [161].

Summarily, these studies indicate that SFN modifies risk factors for CVDs; body weight, lipid profile, blood pressure, blood glucose, serum insulin, and HOMA-IR, which translated to improvement of metabolic syndrome and DM features [61]. SFN prevents atherosclerosis through modulating various pathways involved in oxidative stress, inflammation, and lipid metabolism and also prevents restenosis of arteries. SFN inhibits key events in atherosclerosis development and progression through MAPK/AP-1/NF-κB [146] and Nrf2/ARE pathways and possibly the cPKCβII/Akt (Akt-protein kinase B) signal pathway [131]. SFN improves morphological, histological, and echocardiographic parameters in MI more than mechanical postC, secondary to upregulation of antioxidant response, inhibition of oxidative stress, lipid peroxidation, inflammation, apoptosis and autophagy [62]. In CHF, SFN improves cardiac function, curtails hypertrophy and fibrosis [63], and was able to stop the onset of diabetic cardiomyopathy in a mouse model [157,158]. Cardioprotective effects were also demonstrated against pulmonary hypertension [65], arrhythmia [64], aging [66], and chromium-induced heart toxicity [159].

#### 5.1.2. Phenethyl Isothiocyanate (PEITC)

PEITC is a by-product of the myrosinase hydrolysis of gluconasturtiin, which is found in turnips and radish [48]. PEITC showed potent inhibition of a variety of cancer-promoting processes and is currently being tested in clinical studies for leukemia and lung cancer [47]. PEITC inhibited angiogenesis by causing inactivation of Akt and suppression of proangiogenic growth factor secretion [162]. Similarly, PEITC prevented neovascularization by inhibiting autophagy-mediated angiogenesis and the expression of VEGF in a DM model of retinopathy [163]. The effect of PEITC on CVDs has not been well studied. Nonetheless, available research has shown some degree of cardioprotection. Researchers have reported its ability to alleviate oxidative stress, inflammation [164,165], and apoptosis [82]. PEITC was shown to inhibit ΙκΒ-α degradation, NF-κBp65 nuclear translocation, and binding to DNA (deoxyribonucleic acid), and consequently, NF-κB luciferase activity [78]. Hence, PEITC decreased expression of NO, TNF-α, IL-10 [165], IL-1β, inducible nitric oxide synthase (iNOS), cyclooxygenase-2 (COX-2), and prostaglandin E (PGE) [78,164]. On the other hand, PEITC increased Nrf2/ARE-luciferase activity [166] and similar to structurally related SFN and AITC, PEITC increased ERK1/2 phosphorylation, nuclear Nrf2 translocation, and the expression of γ-glutamyl cysteine synthetase (γ-GCS), HO-1, and NQO-1 [167].

In a dose dependent manner, PEITC greatly reduced body weight gain in high fat diet (HFD)-fed mice compared to control via reducing adipocyte differentiation and inhibiting expression of lipogenic enzymes and mediators [168]. It also reduced food intake by activating leptin signaling; leptin is a hormone that causes a feeling of satiety [79]. PEITC reduced serum levels of insulin, blood glucose, HOMA-IR, and suppressed lipid accumulation. It also reduced atherosclerotic plaque formation (and atherosclerotic index) preventing arterial luminal narrowing by specifically suppressing arterial LDL-C deposition [80]. PEITC caused reverse cholesterol transport via upregulating the peroxisome proliferator-activated receptor gamma (PPARγ)-LXR-α-ABCA1 pathway and inhibited ensuing atherosclerotic plaque inflammation through epigenetic modification of HDAC3 and NF-κB genes [80]. Again, PEITC enhanced epigenetic regulation of Nrf2 expression through H3K4me1 enrichment of the promoter region of Nrf2 gene (NFE2L2) and because of this, it was proposed that PEITC may relieve cardiovascular complication in DM [81]. Some authors recommended that PEITC should be investigated as a HDAC inhibitor for possible use in heart failure [156]. PEITC was able to limit atherosclerotic plaque progression by inhibiting foam cell formation, suppressing internalization of lipids, and enhancing efflux of cholesterol from foam cells [169]. These were mediated through downregulation of lipid receptors’ LOX- 1 (lectin-like oxidized low-density lipoprotein receptor-1), SR-A1 (scavenger receptor A1), and CD36 (cluster of differentiation 36) expression on foam cells and by increasing LXR-α/PPARγ-dependent expression of cellular cholesterol efflux protein (ABCA1, ATP binding cassette subfamily A member 1), respectively. The expression of SIRT1 (sirtuin 1), which blocks NF-κB signaling pathway, was also increased by PEITC [169]. Similar to the structurally related SFN, PEITC protected endothelial cells against oxLDL-induced damage. PEITC blocked NF-κB-induced adhesion molecules production and upregulated Nrf2-dependent antioxidant activity [139]. Specifically, PEITC decreased ROS production, upregulated Nrf2/ARE driven expression (HO-1, GSH, glutamate-cysteine ligase catalytic subunit (GCLC), glutamate-cysteine ligase modifier subunit (GCLM)), downregulated expression of ICAM-1, VCAM-1, E-selectin, NF-κB-p65, p-IκB-α, inhibited adhesion of monocytes to endothelium and therefore protected arteries from endothelial cell injury and atherosclerosis [170]. In Acquired Immuno-Deficiency Syndrome (AIDS) model, PEITC and SFN suppressed Human Immunodeficiency Virus (HIV)-retrovirus-induced upregulation of iNOS and lowered Bax mRNA (messenger ribonucleic acid) expression, and hence drastically prevented left ventricle cells from apoptosis. PEITC also reduced mortality compared to disease group [82].

Though mainly researched for cancer treatment and prevention [47], PEITC has shown some desirable effects in cardioprotection. PEITC may lower the risk of developing heart diseases [80]. It is particularly outstanding in its ability—through various mechanisms—to prevent the onset and halt the progression of atherosclerosis [169], which is known to play a vital role in cardiovascular diseases and complications. Key pathways in CVDs are modulated by PEITC as shown, with epigenetic modulation of some genes that in turn caused suppression of atherosclerotic plaque formation. In addition, PEITC reduced weight gain and alleviated features of DM [81]. Here, we urge more researches in its cardiovascular role.

#### 5.1.3. Moringin (MG)

*Moringa oleifera* Lam. (Moringaceae) is rich in glucomoringin (GMG), an uncommon form of GL found mostly in the seeds of the plant [171,172]. Besides that, the ITC resulting from myrosinase hydrolysis, known as moringin (MG), glucomoringin isothiocyanate (GMG-ITC), or 4(-L-rhamnosyloxy)-benzyl isothiocyanate has shown a broad range of biological activities, such as antibacterial, anti-inflammatory, tumour inhibiting, and apoptosis inducing activities, similar to some other ITCs [36]. GMG has no appreciable anti-inflammatory activity prior to bioactivation [173].

In a couple of studies, MG abolished mRNA expression of the pro-inflammatory mediators iNOS, NO, IL-1β, IL-6, IL-8, TNF-α, COX-2, MCP-1, Toll-like receptor-4 (TLR4), and P-selectin [86,174,175,176,177,178]. MG modulated NF-κB and JAK/STAT (Janus kinases/signal transducer and activator of transcription) signaling pathways and reduced expression of signal transducer and activator of transcription 5 (STAT5) gene in the latter case [176]. MG demonstrated better potency than SFN in activating Nrf2/ARE transcription, increased gene and protein expression, and activity of Nrf2 and its responsive genes (NQO-1, HO-1, and GCLC) [86,178,179]. Furthermore, MOR/α-CD (moringin/alpha-cyclodextrin) complex (as is discussed in Section 5.2.3) inhibited the activation of the MAPK genes Akt and p38, decreased nitrotyrosine and Bax expression, and increased Bcl-2 [177]. A transcriptomic analysis revealed the ability of MG to inhibit a large group of genes that mediate inflammation and oxidative stress including TNF-α, interferon alpha (IFN-α), IL-1β, IL-6 among others, increase Nrf2 gene expression and nuclear accumulation, decrease NF-κB translocation and its binding to promoter sites on responsive genes, decrease ROS production especially hydrogen peroxide (H₂O₂) content of mitochondria, and restore mitochondrial membrane potential. Hence, MG showed drastic suppression of systemic inflammation [180].

*M. oleifera* Lam. seed extract minimized infarct sizes, alleviated myocardial contractile dysfunction, counteracted MI-induced cardiac remodeling/fibrosis, prevented ventricular failure, and provided an overall reduction in mortality post-MI in comparison to disease group [83]. The mechanism was based on the ability of the extract to abolish apoptosis, through arresting mitochondrial apoptotic pathway and reducing production of gp91phox, iNOS, and collagen [83]. On the other hand, the leaf extract demonstrated significant improvement in heart rate, albeit with no demonstrable effect on mean arterial blood pressure (MABP). It caused reduction of preload (decrease LVEDP) and improvement in cardiac performance through improvement of myocardial contraction and relaxation [84]. Further, it suppressed infiltration of poly-morphonuclear leucocytes, myonecrosis, and fibrotic changes induced by doxorubicin with lower cardiac biomarkers’ levels, i.e., LDH, CK-MB (creatine kinase myocardial band), and SGOT (serum glutamic-oxaloacetic transaminase). Demonstrable increment in cardiotonicity with subsequent increase in oxygenated blood supply to the myocardium and other organs, improved electrocardiographic (ECG) features and reduction in mortality from congestive heart failure were recorded [85].

Invariably, the effect of MG was also studied as isothiocyanate-enriched extract or fraction of moringa seeds or leaves either alone or in combination with another moringa ITC. The moringa concentrate contains two major ITCs viz; 4-[(α-L-rhamnosyloxy) benzyl]isothiocyanate and 4-[(4′-*O*-acetyl-α-L-rhamnosyloxy) benzyl]isothiocyanate [174]. Isothiocyanate-enriched moringa extract (containing 47% of the ITC) ameliorated features in DM in C57BL/6J mice that developed obesity and insulin resistance following a 12-week high-fat-diet. It reduced body weight, adipose tissue mass, and blood glucose, in addition to suppression of inflammation and improvement of gut microbiome in the experimental animals [86]. Similarly, the moringa concentrate had anti-obesity and anti-diabetic effects due to the effect of the ITCs in inhibiting rate-limiting steps in liver gluconeogenesis, and increasing insulin signaling and sensitivity. It also increased lean body mass [175].

Concerning MG, few studies of the disease model have been conducted so far. These include studies on ischemic stroke [181], Alzheimer’s disease [90], multiple sclerosis [182], neuroblastoma [183], and human malignant astrocytoma [96]. These shall be discussed under the neuroprotective effect section below (Section 5.2.3), except for ischemic stroke. In ischemic stroke model, MG was able to limit progression of brain damage by downregulating the NF-κB pathway; preventing IκB-α degradation and NF-κBp65 translocation that led to suppression of proinflammatory markers’ (TNF-α, phospho-ERK p42/44, p-selectin, iNOS, MMP-9 (matrix metalloproteinases-9)) expression. Henceforth, MG prevented gait abnormalities [181]. It is worth mentioning that a strategy has been proposed by Fahey et al. [173] on how to deliver “Precise Oral Doses” of MG and other ITCs in human studies. This formulation has been made in the form of cold and hot tea for MG and its precursor GMG, respectively. The anti-inflammatory effect of the tea formulation produced a comparable effect to the reference standard (purified ITC and SFN) used in the case of MG [173].

From the above discussion on MG, it is evident that research on it is mainly related to oxidative stress and inflammation; MG upregulates Nrf2 pathway and downregulates JAK/STAT/NF-κB signaling pathway. The ability of a phytochemical to antagonize these pathways makes it possible to relieve chronic diseases, of which CVDs are enlisted. MG may reduce CVD risk by reducing obesity and DM features [175]. It is through the antioxidative, anti-inflammatory and anti-apoptotic effects that MG relieved ischemic stroke [181]. We urge upcoming researchers in this area to investigate widely other possible protective effects of MG in various CVD models. Researchers have the paved way for clinical studies involving the use of MG by developing a palatable formulation [173]. However, with the knowledge that MG is poorly soluble and stable in water, MG/α-CD complex may also be considered for a similar formulation.

#### 5.1.4. Erucin (ER)

Glucoerucin, the GL precursor of erucin (ER), is present in high concentration in rocket salad species, e.g., arugula (*Eruca sativa*, Mill.), kohlrabi, Chinese cabbage or wild rocket [97,98], and broccoli seeds [184,185]. Myrosinase hydrolysis of glucoerucin produces erucin [1-isothiocyanato-4-(methylthio)butane], which has a similar structure to SFN [97] as its sulfide counterpart [186]. Antioxidant activity of ER has been reported in many studies [185,187,188,189], which was said to be due to radical scavenging activity and/or hydrogen-donating ability [187]. Compared to SFN, it was shown to possess better effect on mRNA induction of phase II enzymes [186].

ER induced ERK1/2-, JNK-, and p38-dependent signal transduction pathways with more potent increase in mRNA and protein levels of nuclear Nrf2 and HO-1 than AITC, SFN, and PEITC in a comparative study. The effect of ER on HO-1 solely depended on induction of the p38-dependent pathway [190]. Another comparative study on the anti-oxidant effect of ER and SFN in rat (Wistar) and human liver slices showed poor upregulation of NQO-1 activity in human liver compared to rat liver and these ITCs upregulated different isoforms of GST enzyme in rat and human liver [51]. In other studies, ER improved the activity and raised the expression of GST and quinone reductase (QR) [41,191]. Concerning the NF-κB pathway, ER inhibited IκB-α degradation and p65 nuclear translocation, suppressed NF-κB DNA binding/transcriptional activity causing decreased expression of TNF-α, IL-6, IL-1β, iNOS, and COX-2, as well as activity of iNOS and COX-2 [192].

ER reduced intracellular lipid accumulation by inhibiting expression of adipocyte marker proteins/adipogenic genes; PPARγ, CCAAT/enhancer-binding protein alpha (C/EBPα), fatty acid synthase (FAS), and sterol regulatory element binding protein-1c (SREBP-1c) and Raf/MEK/ERK/p90RSK pathway [193]. In a recent study, *E. sativa* seed extract reduced weight gain, body mass index (BMI), TG, fasting blood glucose, and HbA1c levels as well as reducing adipose tissue weight/mass, increasing metabolic activity of adipose tissue, and decreasing adipocyte size [99]. Further, *E. sativa* extract/fraction (given intravenous and oral) reduced MAP in hypertensive rats; effect became nullified by pretreatment with atropine, hence authors proposed its action on muscarinic receptors (stimulate NO release, inhibit Ca2^+^ influx/release). The extract/fraction induced endothelium-independent relaxation of aorta and promoted negative inotropic and chronotropic effects on heart atria [194]. In human aortic smooth muscle cells (HASMCs), ER caused the emission of H₂S, which in turn induced hyperpolarization of the membranes of HASMCs. ER made rat aortic rings to dilate and even though it had shown no effect on basal coronary flow, it recovered the flow in precontracted coronary arteries to normal [100]. ER reduced systolic blood pressure (SBP) in spontaneously hypertensive rats (SHRs) by roughly 25% and restored blood pressure to normotensive rats’ levels [100]. ER preserved the integrity of endothelial wall and stimulated intracellular release of H₂S in HUVECs also [101]. In the setting of hyperglycemia, ER prevented ROS production, downregulated expression of inflammatory mediators (NF-κB, NADPH oxidase p22phox, COX-2, TNF-α and IL-6), inhibited caspase 3/7 activation, averted endothelial hyperpermeability, and preserved endothelial tight junction function (increases expression of vascular endothelial-cadherin (VE-Cadherin) and zonula occludens-1 (ZO-1) proteins, which are cell-cell contact proteins) [101]. *E. sativa* extract also showed antiplatelet and antithrombotic activities: It inhibited activation of NF-κB p65 in platelets and the expression of P-selectin, thromboxane B2, CCL5 (chemokine(C-C motif) ligand 5), transforming growth factor beta (TGF-β) and IL-1β that contributed to suppression of platelet aggregation and reduction in arterial thrombus formation [195]. On the heart, *E. sativa* extract prevented cardiac toxicity as it inhibited elevation in cardiac markers (CK-MB, LDH, and myoglobin), increased anti-oxidative enzyme levels (SOD, CAT, GSH), reduced thiobarbituric acid reactive substances (TBARS) synthesis, inhibited expression of p53, and prevented histological alteration of the myocardium [102].

Taken together, ER has shown activities of a cardioprotective agent. Studies highlighted have revealed stimulation of MAPK/Nrf2 pathway and inhibition of NF-κB pathway by ER and/or *E. sativa* [190,192]. Those effects explain ER’s role in maintaining endothelial integrity [101]. ER suppressed lipid accumulation, weight gain, obesity, and stabilized biochemical features of DM [99]. ER preserved myocardial viability and shielded the myocardium from toxicity [102]. Like other ITCs, the pharmacological effect of ER has been linked to H₂S release, which may explain protection of endothelial integrity and reduction of SBP by ER [100,101].

#### 5.1.5. Allyl Isothiocyanate (AITC)

Wasabi (*Wasabia japonica* Matsum) is a native Japanese plant that contains appreciable amounts of allyl isothiocyanate (AITC), a compound resulting from sinigrin (GL precursor) hydrolysis [104]. AITC has reportedly been identified as a possible therapy for obesity and insulin resistance. According to studies, AITC treatment can prevent HFD and palmitic acid-induced lipid accumulation and inflammation via combined Sirt1/AMPK pathway upregulation and NF-κB pathway downregulation [196]. Other authors discovered that AITC inhibited the activity of the sterol regulatory element (SRE)-containing FAS promoter in human hepatoma Huh-7/FAS-luc cells reducing SREBPs target gene expression as well as de novo fatty acid and cholesterol synthesis. In addition, AITC was shown to promote a drop in body weight and blood glucose levels in rats suggesting a new physiological role of AITC in lipid metabolism regulation [197]. Metabolic dysregulation resulting from adipocyte hypertrophy, which promote the development of CVDs and their risk factors, has been shown to be markedly suppressed by Wasabi leaf extract (WLE) through inhibiting expression of genes involved in fat accumulation including; PPARγ, hepatic acetyl-CoA carboxylase 1 (ACC1), adipose tissue leptin, C/EBPα, C/EBPβ, FAS, SREBP1c, and hepatic AMPKα1 and AMPKα2 [104,198]. WLE reduced weight gain, elevation of blood pressure, serum glucose, insulin, hemoglobin A1c (HbA1c), TG, total cholesterol, and low-density lipoprotein (LDL) levels, and increased serum HDL-C level [104,198]. On the other hand, WLE enhanced lipolysis and lipid metabolism by increasing β3-adrenergic receptor (Adrb3) mRNA expression [199] and adiponectin expression via activating AMPK/ACC pathway [104]. A more recent study showed that WLE can decrease both total body and abdominal fat densities [198]. WLE suppressed HFD-induced lipid peroxidation by normalizing content and activities of GSH, CAT, and SOD [198], and also inhibited NO production and release in LPS-activated macrophages [200].

In models of obesity, AITC reduced body weight, blood glucose, serum TG, and fat accumulation by inhibiting adipocyte differentiation through downregulating adipogenic transcription factors (C/EBPα, C/EBPβ, PPARγ, LPL (lipoprotein lipase), FAS, aP2 (adipocyte protein 2), and adipsin), galectin-12 expression, and adipokine expression (leptin and resistin). Furthermore, AITC inhibited activation of Akt-mTORC1 (mammalian target of rapamycin complex 1) and CREB (cyclic adenosine monophosphate (cAMP) response element-binding protein) and decreased NF-κB expression responsible for inflammation [201,202]. AITC treatment for ten weeks in HFD-fed mice reduced diet-induced hyperglycemia and weight gain, normalized serum lipids, and alleviated insulin resistance by causing an increase in mitochondrial membrane potential and mitochondrial DNA content [105]. Also, AITC was reported to modulate lipid metabolism via increasing the expression of proteins involved in fatty acid β-oxidation [196], while lowering blood glucose follows increased glucose utilization through activating transient receptor potential vanilloid 1 (TRPV1) channels in mice [203]. By activation of transient receptor potential ankyrin 1 (TRPA1) channel associated with release of CGRP (calcitonin gene-related peptide), AITC dilated dural and pial arteries and lowered MABP in experimental rat model of migraine [106]. AITC activated Ca^2+^-permeable nonselective cation channels in cardiac fibroblasts probably via TRPA1 as it is a selective TRPA1 agonist. This increased Ca^2+^ influx and its intracellular concentration [204]. Some researchers have developed a TRPA1 biomarker assay based on dermal blood flow measurement for future use in certain disease models and human clinical trials (neuropathic pain, inflammatory skin conditions, asthma, and chronic cough) [205].

AITC suppressed pro-inflammatory cytokines such as IL-1β, IL-6, and TNF-α expression in HUVECs [206,207]. AITC nanoparticles used in the study of Chang et al. [208] suppressed TNF-α, IL-6, NO, and iNOS production even more [208]. AITC decreased iNOS expression and reduced microRNA-155 (miR-155) levels (belongs to microRNAs class of transcription factors involved in transcriptional regulation of cellular processes including inflammation) by inhibiting NF-κB p65 nuclear translocation [209]. AITC was demonstrated to directly bind to lysine and arginine amino acids on the NF-κB gene forming hydrogen and hydrophobic bonds, which may partly explain suppression of transcriptional activity of NF-κB [207] alongside inhibition of JNK, ERK, and p38 phosphorylation [210]. AITC increased ERK1/2 phosphorylation, nuclear Nrf2 translocation, and the expression of Nrf2 responsive genes; γ-GCS, HO-1, and NQO-1 [167,209]. Similar to its effect on NF-κB gene, AITC stimulated AhR and Nrf2 through hydrophobic and hydrogen bond interactions and raised the synthesis of phase II enzymes, increased antioxidant activity, and reduced lipid peroxidation [211]. Moreover, AITC caused SKN-1 (skin head-1)-induced expression of GST-4 and UGT-13 (UDP-glucuronosyl/glucosyl transferase-13); which conferred resistance to oxidative stress and increased lifespan in a nematode model [212].

With those beneficial effects of AITC, it is not without adverse effects (see Section 6 for GLs and ITCs adverse effects) as inhalation of AITC in normotensive and SHRs resulted in bradycardia, atrioventricular (AV) block, prolonged PR intervals, and biphasic blood pressure response. The latter effect was explained as a brief hypertensive phase followed by a hypotensive phase occurring secondary to TRPA1 channel activation associated with stimulation of autonomic sensory neurons [213]. In SHRs alone, AITC caused abnormal electrocardiogram (ECG) pattern with increased occurrence of negative P and duration of P waves, and also increased PR and RR intervals than in controls [214].

The main roles of AITC in CVD protection include obesity reduction and lowering metabolic syndrome features, in addition to lowering oxidative stress and inflammation. AITC suppresses inflammation and oxidative stress by modulating MAPK/NF-κB [210] and AhR/Nrf2 pathways, respectively [211]. AITC reduces weight gain by both enhancing lipolysis and preventing lipid deposition [196]. The increase in Ca^2+^ influx as reported by Oguri et al. [204] may increase myocardial contractility in heart failure and MI; this hypothesis has to be tested in an in-vivo study. However, caution is needed in clinical trials involving heart disease patients. Therefore, the inhalational route might not be a good gateway for AITC treatment for patients with background heart disease and hypertension [214].

#### 5.1.6. Indole-3-Carbinol (I3C)

Indole-3-carbinol (I3C) is derived from glucobrassicin through myrosinase hydrolytic reaction. After formation, it undergoes self-condensation reaction to form 3,3′-diindolylmethane (DIM) [215]. In CVD protection, DIM and I3C modulated cellular pathways of inflammation, oxidative stress, autophagy, and angiogenesis; principally, PI3K/Akt/mTOR, MAPK, NF-κB, and AhR/Nrf2 signaling pathways [215]. Apart from its well-known chemopreventive effects, I3C has demonstrated anti-obesity, anti-diabetic, and cardioprotective effects in many studies [215,216]. However, some researchers ascribed the effect of I3C to its metabolite DIM in a study that reported prevention of cardiac cell hypertrophy by downregulation of AMPKα and MAPK/mTOR signaling pathway [217].

Earlier research concentrated on the chemopreventive effects of I3C: when compared to the solid Ehrlich carcinoma group, DOX and/or I3C resulted in a considerable reduction in tumour volume [218]. In the same study, I3C prevented the cardiotoxic effects of the anticancer drug and supplemented cardiac antioxidant status (increased cardiac CAT and SOD, decreased MDA) [218]. According to Deng et al. [108], I3C’s effect on apolipoprotein B (apoB) synthesis, as well as its antiplatelet and anti-thrombotic abilities are capable of attenuating cardiac remodeling through enhancing energy metabolism. Again, Deng et al. [219] showed protection of pressure overload-induced cardiac remodeling by I3C through activation of AMPK enzyme, resulting in improved cardiac functioning and reduced hypertrophic and fibrotic marker gene expression. Therefore, it may be useful in heart failure treatment [219]. Hajra et al. [220] showed that I3C may prevent cardiotoxicity through increasing GSH and other phase II enzymes’ levels, suppressing oxidative/nitrosative stress and lipid peroxidation, upregulating Nrf2/ARE pathway, downregulating NF-κB pathway (NF-κBp50, iNOS, COX-2, and IL-6), and balancing apoptotic markers’ expression (Bcl2, casp3, Bax). I3C stimulated muscarinic M2 receptors in the heart heralding parasympathomimetic response and abolishing cardiac hypertrophy, and a rise in heart rate and blood pressure. I3C also increased cardiac NO level, normalized serum myocardial markers CK-MB and LDH, and reduced myeloperoxidase and hydroxyproline expression [109].

Furthermore, I3C alleviated biochemical alterations in DM model. Among features suppressed by I3C include blood glucose, insulin, hemoglobin, HbA1c, markers of oxidative stress, and lipid peroxidation. The effect of its metabolite DIM was greater than that of the standard drug used in the experiment [110]. The ability of I3C to lower serum cholesterol levels in hypercholesterolemic mice has been reported by Maiyoh et al. [221]: I3C was shown to significantly inhibit hepatic apoB-100 production leading to the alteration of cellular lipid synthesis. One of the methods for I3C’s inhibition of apoB secretion was reduced lipid synthesis via SREBP-1, which modulates cholesterol homeostasis, and its downstream gene, FAS [221]. I3C prevented body weight gain, obesity, and inflammation as a result of obesity by modulating essential pathways involved in adipogenesis, thermogenesis, and inflammation. I3C decreased pro-inflammatory cytokines expression and levels of serum glucose, TG, insulin, and leptin, but increased serum adiponectin concentration. Furthermore, I3C inhibited macrophage recruitment to adipose tissue, decreased fatty acid synthesis, decreased proliferation and survival of adipocytes, inhibited lipid accumulation, and promoted lipid metabolism [222,223,224,225,226]. In mature adipocytes, the inhibition of lipid accumulation resulted in increased expression of AhR and cytochrome (CYP1B1) proteins, and slightly reduced expression of Nrf2, hormone-sensitive lipase, VEGF receptor, and glycerol-3-phosphate dehydrogenase. Importantly, I3C inhibited endothelial tube formation by modulated secretion of VEGF, IL-6, matrix metalloproteinases, and NO in adipocytes [227]. Also, I3C inhibited lipid deposition in blood vessels; it promoted autophagy in hyperlipidemia zebrafish model with modulation of class III PI3K/Akt/mTOR pathway [111].

Conclusively, I3C can reduce pressure overload effect and protect the heart from hypertrophic changes [109]. It is evident that I3C modulates intricate pathways in preventing obesity [222,223,224,225,226]. By suppressing lipid deposition in blood vessels [111] and inhibiting endothelial tube formation [227], I3C may prevent atherosclerosis. I3C balances both biochemical parameters in blood/serum [110] and genetic expression of relevant genes involved in cardioprotection [220]. The beneficial effects of ITCs (SFN, *M. oleifera*, *E. sativa*/ER, I3C) on CVDs with proposed underlying mechanisms of action are summarized in Table 2 below.

### 5.2. Neuroprotective Effect

By the 2040s, neurodegenerative disorders (NDDs) are expected to overtake cancer as the leading cause of mortality [60]. Neurons—the building blocks of the CNS—are incapable of reproducing or replacing themselves. Once the damage is imminent, cell loss will be permanent [60]. Misfolded proteins, oxidative stress, inflammation, mitochondrial dysfunction, excitotoxicity, and, of course, neuronal death are common features of various diseases such as Alzheimer’s disease (AD), Parkinson’s disease (PD), Huntington’s disease (HD), amyotrophic lateral sclerosis (ALS), i.a. [4,5,228,229,230]. Now, natural products from plants and/or animals are vigorously researched into as potential therapeutics for NDDs, as current drugs have so far not been very effective in treating such diseases [5,8,231,232]. A recent review has pointed out the role and effectiveness of plant-based medicines due to their phytochemical constituents [231]. In order to curtail these disorders, several studies have been conducted on the use of GLs and ITCs with significant outcomes as will be discussed hereunder. ITCs were reported to play protective roles in both acute and chronic NDDs [229].

#### 5.2.1. Sulforaphane (SFN)

Oxidative stress is involved in the pathogenesis of many NDDs such as AD, PD, and HD [8]. SFN showed protection against the progression of these diseases by significantly lowering oxidant stress in the brain [229]. In primary neuronal cultures of rat striatum, SFN prevented oxidative stress-induced cytotoxicity in a dose-dependent manner [233]. Gene profile analyses revealed that most of the genes induced by SFN are linked to the oxidative stress response and to some extent those involved in metabolism of glutathione and xenobiotics by cytochrome P450 [234]. The protective effect of SFN on acute and chronic neurodegeneration was analyzed by Tarozzi et al. [229], where they pointed out that SFN’s activation of Nrf2 pathway serves the basis for anti-oxidant response in ischemic/traumatic brain injury, AD, and PD. SFN promoted anti-inflammatory activity of microglia; induced the Mox phenotype of microglia necessary for antioxidant response via ERK/Nrf2 pathway activation [235]. Further, SFN reduced the expression of proinflammatory mediators, known to contribute to neuroinflammation, within microglia (BV-2 cells) and by extension reduced neuronal loss [236]. SFN downregulated MAPK/NF-κB pathway [236,237], reduced caspase 3 activity/expression, activated Nrf2 in microglia, and led to improvement of amnesic features in mice that were pretreated with SFN-enriched broccoli sprouts [237].

AD is one of the commonest NDDs, accounting for 60–80% of dementia, and mostly affects the elderly population [228,231]. Some authors stressed the need for substances with multiple mechanisms of action since AD is of multifactorial aetiology (amyloid-β (Aβ) cascade, protein misfolding, tau hyperphosphorylation, inflammation, gene mutation, mitochondrial dysfunction, and oxidative stress) and here plants offer a huge diversity of phytochemicals, which may address multiple aetiologies [231]. Importantly, ITCs inhibited cholinesterase activity and showed promising effects in various in vitro and in vivo AD models [231] as will be discussed here. SFN improved clinical features of AD like cognition, memory, and locomotion as observed in behavioral tests. It reduced cognitive impairment in passive avoidance and retention tests (*p* < 0.01) [238,239], and in open field and Morris water maze tests [67]. SFN was able to counteract intracerebroventricular (ICV) injection of Aβ aggregate-induced memory deficit in mice and improve both spatial and contextual memory in Y-maze test [238], while also improving locomotor activity [68]. Through multiple mechanisms explained below, SFN prevented cognitive impairment in experimental models of AD by reducing biomarkers of Aβ, tau, inflammation, oxidative stress, and neurodegeneration [240]. SFN prevented cholinergic neuron death in the medial septal and hippocampal CA1 areas, which led to reduction of cognitive impairment in AD-like lesion rats [239]. In mice exposed to scopolamine-induced memory impairment, SFN increased acetylcholine (ACh) level, decreased acetylcholinesterase (AChE) activity, and increased choline acetyltransferase (ChAT) expression in the hippocampus and frontal cortex [241]. A study by Kim et al. [238] shed light on neuroprotective effects of SFN in an acute AD mouse model [238]. In order to facilitate the initial learning and memory deficiency, the mice were subjected to a single ICV injection of Aβ aggregates, then administered SFN via intraperitoneal (IP) injection for six days. Although it did not directly interact with Aβ, SFN reduced cognitive impairment and therefore proposed to protect the brain against amyloidogenic damage [238].

It was thought previously that SFN does not regulate amyloidogenesis because thioflavin T (ThT) assay and transmission electron microscopy (TEM) imaging revealed that SFN does not inhibit ThT-Aβ interaction nor Aβ aggregation [238]. However, SFN was found to reduce production and deposition of Aβ plaques in hippocampus and cerebral cortex of AD-lesion and transgenic AD mouse models and the associated neurobehavioral deficit [67,68], as well as to suppress neuronal death in Aβ-exposed human neuroblastoma cell line (SH-SY5Y) [67]. Also, SFN inhibited production of Aβ oligomer and aggregation of Aβ [69], protected against AD-induced oxidative and carbonyl stress [68], and suppressed cytotoxicity and apoptosis in SH-SY5Y cells exposed to Aβ_25–35_-induced cytotoxicity [242]. SFN increased expression of CHIP (C-terminus of HSP70-interacting protein) and its co-factor heat shock protein 70 (HSP70), which in turn reduced protein levels of monomeric and polymeric forms of Aβ and inhibited tau aggregation [243]. In addition, SFN epigenetically modified Nrf2 by decreasing DNA methylation levels of the Nrf2 promoter gene and led to increased Nrf2 expression, translocation, and activity. Similar epigenetic modification decreased NF-κBp65 activation. the combined effect on these genes led to improvement in antioxidative and anti-inflammatory activity in AD with reduction in Aβ production [244]. Further, SFN reduced Aβ burden by promoting activity of processes involved in Aβ degradation. Specifically, SFN increased acetylation of histone H3 and H4 at lysine K9 and K12, respectively, thereby increased levels of Ace-H3K9 (acetylated histone 3 lysine 9) and Ace-H4K12 (acetylated histone 4 lysine 12) led to decreased mRNA and protein expression of HDAC1, HDAC2, and HDAC3 and hence upregulation of p75 neurotrophin receptor (both its MRNA and protein expression) [67]. SFN’s effect on blocking the NF-κB pathway and rise in intracellular Ca^2+^ levels—both of which can cause a rise in cytokines (IL-1β and TNF-α) that inhibit Mer tyrosine kinase (MerTK) expression—caused increased expression of MerTK, which in turn stopped neuroinflammatory processes secondary to Aβ in a negative feedback mechanism [245]. On the other hand, SFN reduced microRNA-146a in the AD brain and IL-1β production in microglia by downregulating STAT-1 activation and upregulating Nrf2/HO-1 signaling pathway. This resulted in decreased Aβ peptide-induced-cathepsin B- and caspase-1-dependent NLRP3 inflammasome activation and hence inhibited activation of STAT-1 as well as reduced IL-1β production [246]. SFN also suppressed Aβ-induced reduction of microglial phagocytic activity [247]. Similarly, the protective role of SFN has been investigated by Park et al. [248], who reported the effectiveness of SFN in causing Aβ degradation by upregulating luciferase activity and expression of the catalytic subunit of proteasomes PSMB5 and PSMB6 in murine neuroblastoma Neuro 2A cells exposed to Aβ_1–42_-induced cytotoxicity. This enhanced proteasome function and helped cells resist Aβ_1–42_-induced cytotoxicity better than untreated cells [248]. SFN also attenuated proteasome inhibition (that occurs after birth) and improved proteasome activity as well as hippocampal development and spatial learning and memory in day-old mice exposed to ICV injection of MG132 (Z-Leu-Leu-Leu-al) [249].

In PD, SFN has been shown to confer neuroprotective effect through various pathways as demonstrated in many studies with good improvement of behavioral and motor symptoms in experimental animals, shouldering neurons from toxic effects of hydrogen peroxide (H₂O₂), 6-hydroxydopamine (6-OHDA), and 5-S-cysteinyl-dopamine (CysDA) through suppressing oxidative stress and arresting apoptosis [70,71,250]. The linkage between oxidative stress and pathogenesis of PD is well known and models of the disease depict this feature. The effect of SFN in PD centers on the reduction of oxidative stress, alongside protection of dopaminergic neurons from lipid peroxidation preserving their cell membranes and consequently leading to reduction in neuronal apoptosis via the mitochondrial apoptotic pathway [251]. In the latter case, SFN inhibited DNA fragmentation and caspases 9 and 3 expression and activities [71]. A study has shown 76% and 78% reduction in apoptosis in H₂O₂ and 6-OHDA models, respectively [250]. In primary cultures of mouse cortical neurons exposed to 5-S-cysteinyl-dopamine induced neuronal injury, SFN (0.01–1 µM) was found to be effective in preventing cellular apoptosis in a dose-dependent manner with peak effect at 100 nM [70]. SFN was also able to reduce oxidative stress through modulating the PI3K/Akt/ERK1/2 and Nrf2/Keap1 signaling pathways with consequent increase in the level of phase II anti-oxidative enzymes. SFN reduced ERK1/2 phosphorylation [70,71,252], but the effect of SFN in PD was mainly correlated with the GSH activity and not the other anti-oxidative enzymes (CAT, SOD) [250]. SFN increased intracellular GSH content to about 125 mmol/μg total protein in a concentration and time-dependent manner when administered at a dose of 2.5–5 μmol/L for 24 h and it increased the expression and activities of all the glutathione-related enzymes, i.e., glutathione reductase (GR), GST isoenzymes except GSH-Px [70,71,250]. Therefore, SFN increased total antioxidant capacity of the cytosolic component of SH-SY5Y cells [250]. SFN also induced QR1 gene expression and increases its activity [251], but had no effect on JNK and p38 [70]. Through relieving oxidative stress, SFN also preserved neuronal length [251] and besides reducing cytotoxicity through the mitochondrial apoptotic pathway [251], it reduced cytotoxicity from 6-OHDA-induced endoplasmic reticulum (ER) stress (inhibited expression of Bip (an ER chaperone) and CHOP (C/EBP homologous protein)) in rat PC12 cells by increasing translocation of Nrf2 to the nucleus [253].

Different mechanisms have been highlighted on dopaminergic (DAergic) neuronal protection by SFN. SFN has been shown to increase the expression of tyrosine hydroxylase (TH) in the substantia nigra [71] and ventral midbrain [254] of 6-OHDA and 1-methyl-4-phenyl-1,2,3,6-tetrahydropyridine (MPTP) models of PD, respectively. Also, in the substantia nigra, there was enhanced neurotrophic factors’ (GAP-43, NGF and BDNF) release, preservation of dopamine transporter, and reduction of cytosolic GFAP expression [255]. SFN prevented tetrahydrobiopterin (BH4)-induced DAergic cell death in SK-N-BE(2)-C, CATH.a, and primary cultured DAergic neurons via removal of dopamine quinone from neuronal cells and decreasing formation and accumulation of protein-bound quinone products in DAergic cells [251]. In addition to its anti-oxidant and anti-apoptotic mediated neuroprotection in PD, SFN inhibited pro-inflammatory effects of MPTP; reduced IL-6 and TNF-α proteins. The anti-inflammatory activity of SFN being through astrocytes of the basal ganglia and not through DAergic neurons nor microglia, even though in addition to reducing the degree of astrogliosis, it also suppressed microgliosis [254].

Moreover, SFN has shown therapeutic effect in ischemic stroke by reducing volume of infarct issued from middle cerebral artery occlusion (MCAO), more pronounced at 5 mg/kg dose and when administered within 15 min after onset of ischemia, as SFN was able to cross the blood–brain barrier (BBB) within a short time [72]. SFN significantly lowered ICAM-1 expression and reduced both recruitment of neutrophils and lymphocytes and degranulation of mast cells around infarcted brain area via downregulating the NF-ĸB pathway [256]. Further, SFN markedly suppressed post-ischemic inflammation (inhibited rise of IL-1β and mature IL-18, decreased NLRP3 inflammasome mRNA level and protein expression) and apoptosis (suppressed levels of cleaved caspase-1) [257]. SFN preserved BBB integrity by modulating the Keap1/Nrf2/ARE, MAPK, and mitochondrial apoptotic pathways. Its effect on inhibition of claudin-5 and P-selectin, and GFAP and Iba-1 expression helped preserve the endothelial lining of the brain and reduce astrogliosis, respectively [258]. In rats pretreated parenterally with SFN prior to left common carotid artery ligation and hypoxia (8% oxygen at 37 °C), a model for neonatal hypoxic-ischemic (HI) injury, SFN reduced infarct ratio and the extent of morphological change of neurons in the cerebral cortex and hippocampus through suppressing lipid peroxidation and apoptosis compared to HI rats that received only vehicle. SFN reduced DNA fragmentation—as evidenced by lower number of TUNEL (terminal deoxynucleotidyltransferase-mediated UTP end labeling)-positive cells—and activity of caspase 3 [73]. SFN minimized the extent of microgliosis (reduced number of Iba1-positive cells and 8OHdG-positive cells) in both grey and white matter areas of the brain through increased expression of Nrf2 protein, with subsequent increases of HO-1 mRNA and protein level in both neurons, astrocytes, and blood vessels [72,73]. SFN lowered 8OHdG level, a marker of DNA/RNA oxidation and subsequent death of astrocytes following oxygen and glucose deprivation (OGD) [259]. SFN also reduced brain histoarchitectural distortion as a result of inflammation [260].

Chang et al. [261] have evidenced that SFN prevents motor neuron death and preserves the number and length of motor neurons in amyotrophic lateral sclerosis (ALS) through activation of the Nrf2/ARE pathway. The combination of SFN, a potent phase II enzyme inducer and riluzole, a classic anti-glutamate agent, produced a more superior effect than either used alone [261]. The antioxidant-mediated effect was stressed by Silva et al. [262] as the most important path to prevention and treatment of ALS [262]. SFN reduced the severity and incidence as well as improved the clinical score of multiple sclerosis, as shown in the disease experimental animal model. It actually reduced by about 40% the development of experimental autoimmune encephalomyelitis (EAE), delayed appearance of symptoms compared to control group by up to four days and lessened clinical disease score in C57BL/6 mice, which received immunization with myelin oligodendroglial glycoprotein peptide (MOG35-55) in complete Freund’s adjuvant (CFA) [74,263]. SFN diminished the extent of autoimmune inflammation in the spinal cord by reducing autoimmune inflammatory infiltrates and demyelination of the spinal cord, hence reduced histological scores [74]. Downregulation of JNK//ERK1/2/NF-κB signaling also improved Treg (regulatory T cell) responses with suppressed Foxp3 expression, while stimulating of Nrf2/ARE pathway reduced the oxidant specie MDA in the brain and overall, neuronal apoptosis was suppressed [263,264,265]. SFN preserved as well the integrity of the blood–brain barrier (BBB) through restoration of claudin-1, claudin-3, claudin-5, and ZO-1 expression and abolishing MMP-9 expression along the meninges and around blood vessels in the CNS [263]. In prion disease model, SFN abolished neurotoxicity through inducing autophagy in human neuron cells exposed to prion protein (PrP) (106–126)-mediated neurotoxicity characterized by increased autophagy flux marker MAP1A/LC3II protein levels, and decreased p62 (an autophagy adaptor protein) levels via activating the AMPK pathway; this was, however, blocked by autophagy-related 5 (ATG5) protein knockdown [266].

In the treatment of schizophrenia, it is known that dopamine enhances oxidative stress in the brain of schizophrenic patients by increasing TBARS and protein-bound quinones and unfortunately, haloperidol, risperidone, and paliperidone all ramp up the oxidative stress induced by dopamine. Interestingly, SFN pretreatment of human DAergic neuroblastoma cells (SK-N-SH cells) exposed to these anti-psychotics abolished lipid peroxidation (decreased TBARS), inhibited accumulation of protein-bound quinones, and curtailed cell death through elevating total GSH levels and increasing antioxidant enzyme activity of NQO-1 and GST [77]. In epilepsy, SFN stimulation of Nrf2/ARE pathway suppressed oxidative stress, progression of amygdala kindling, and cognitive impairment due to seizures [75]. In depression, SFN improved clinical features while biochemical tests revealed reduced corticosterone, adrenocorticotropic hormone, and some cytokines, which made authors propose modulation of hypothalamic-pituitary-adrenal axis and inflammatory response to stress as mechanisms behind this effect. The protective effect was demonstrated in both acute and chronic models [76].

Neuroprotective effect of SFN has cut across various NDDs as discussed in the foregoing paragraphs. SFN ameliorated signs and symptoms and suppressed pathogenetic mechanisms in AD [238,239], PD [70,71,250], ischemic stroke [72], ALS [261], multiple sclerosis [74,263], prion disease [266], epilepsy [75], depression [76], and even curtailed adverse effects of drugs used in schizophrenia [77]. These effects are mainly due to modulation of intricate pathways involved in cellular metabolism and survival as discussed [70,71,245,246,252]. Aβ and tau are the pathogenetic elements in AD; we have seen how SFN decreased Aβ and tau induced brain inflammation, oxidative stress, and apoptosis through multiple mechanisms ranging from reduction in production and aggregation to inhibition of activity [69]. SFN improved cholinergic neurotransmission and clinical features of AD [238]. In PD, SFN was able to increase TH production, reduce protein bound quinones and ultimately preserve the viability of DAergic neurons; these translated to improvement of neurobehavioral symptoms [70,71,250]. Due to fast BBB penetration, SFN reduced inflammatory processes during reperfusion in no time, suppressed neuronal loss, and confined the area of infarct in cerebral ischemia reperfusion models [72].

**Figure 5:**
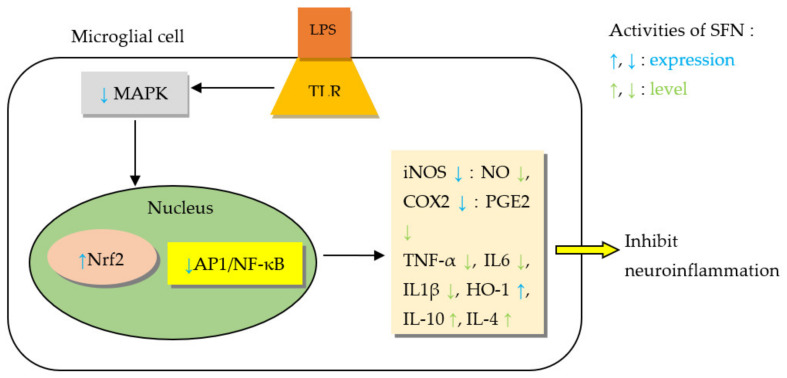
Model of ITCs’-mediated anti-inflammatory activity through inhibition of the JNK/AP-1/NF-κB pathway and activation of the Nrf2/HO-1 pathway, with concomitant upregulation of IL-10 and IL-4. Adapted from Subedi et al. [237].

The inhibition of neuroinflammation by ITCs via inhibition of the JNK/AP-1/NF-κB pathway and activation of the Nrf2/HO-1 pathway is depicted in Figure 5.

#### 5.2.2. Phenethyl Isothiocyanate (PEITC)

Similar to MG and AITC, PEITC has been shown to promote neurogenesis; it promoted dorsal column repair in a model of spinal cord injury through modulating miR-17-5p/STAT3/GAP-43 pathway. PEITC inhibited miR-17-5p, which led to upregulation of STAT3 and GAP-43 expression [267]. AChE activity assay of ITCs in AD showed superior enzyme inhibition from PEITC over benzyl ITC and AITC [39]. Both in vitro and in vivo (LPS-stimulated peritoneal macrophages/ BALB/c mice), PEITC downregulated serum NO and inhibited its release from stimulated LPS-macrophages [268]. The inhibition of Akt activation by PEITC caused suppression of IFN-γ-induced NO production, which led to anti-inflammatory effect [269]. The protective effect of PEITC followed modulation of the MAPK pathway [270]. PEITC reduced the protein levels of (activated) p-p38, p-JNK1/2, p-ERK1/2 proteins, p-AKT serotype Thr308, but increased p-AKT serotype Ser473 and PCNA [270]. Moreover, PEITC directly suppressed IκB α/β (IKKα/β) phosphorylation, IκB-α phosphorylation, NF-ĸB, and p65 protein expression, as well as the subsequent nuclear translocation of p65 and NF-κB, thereby preventing NF-ĸB p65 binding to DNA [270,271]. PEITC upregulated JNK/Nrf2/ARE-dependent expression of phase II enzymes [166] and similar to other ITCs, PEITC’s stimulation of JNK activity was dependent on the presence of GSH [272]. PEITC covalently modified MIF (macrophage Migration Inhibitory Factor), making it unsuitable for antibody binding during inflammatory processes, which made authors propose employment of PEITC as MIF inhibitor to tackle cancers and other inflammatory diseases [273]. Anti-inflammatory effect of PEITC also depended on modulation of Toll-interleukin-1 receptor domain-containing adapter inducing interferon-β (TRIF)-dependent signaling pathway of Toll-like receptors [274].

#### 5.2.3. Moringin (MG)

As mentioned earlier, moringin (MG) is derived from *M. oleifera* Lam. (Moringaceae) plant. Different extracts of this plant have demonstrated neuroprotective effects in models of NDDs [275]. Axonal and dendritic outgrowth and maturation are important steps to establish an efficient neuronal signaling network [87]. Early in neurogenesis, *M. oleifera* extract (MOE) upregulated neuronal differentiation with development of multipolar primary processes, increased length and branching of neurites, and very importantly, protected neurites from naturally occurring cellular injury and improved their viability [87]. In addition, the extract protected the brain from neurodegeneration with reduced degree of cellular atrophy, increased expression of neuron specific enolase (NSE), decreased glial fibrillary acidic protein (GFAP), and also caused diffused expression of enolase-2 throughout the cortical layers [276]. The extract showed high degree of safety in experimental animals with median lethal dose over 5000 mg/kg body weight [276]. MOE improved neurological score in focal ischemia animal model of ischemic stroke with appreciable improvement of both motor and sensory functions by reducing the volume of brain infarct and normalizing levels and activities of MDA and SOD in the cortex and sub-cortex. It arrested MCAO-induced decrease in GSH-Px activity in the hippocampus [88]. In AD, long term (12 weeks) consumption of *M. oleifera*-supplemented diet as powdered leaves mixed with normal rat food suppressed oxidative stress as well as lipid peroxidation by increasing GSH and CAT activity. At the end of the experiment, neurocognition was significantly enhanced, AChE activity was reduced and hippocampal neurodegeneration was suppressed [277]. Following ICV infusion of colchicine, MOE treatment for one week (in addition to the same treatment duration before insult) caused improvement in radial arm maze (RAM) test as well as normalization of catecholamine (norepinephrine, dopamine, serotonin) levels and electroencephalograph (EEG) pattern in the brain of colchicine-exposed rats [89]. EEG showed reduction in spike discharge pattern, increased beta waves, but did not change alpha waves. The extract also reduced the degree of neuronal loss in the cerebral cortex, hippocampus, and the caudate nucleus [89]. Again, when supplemented in mice feed (MO-SD) for 14 days, a great improvement in spatial memory was recorded. In addition to suppressed oxidative stress (decreases MDA and nitrite, increases SOD) and inflammation (decreases TNF-α), MO-SD improved cholinergic neurotransmission by reducing AChE activity, chromatolysis, and loss of cortico-hippocampal neurons [278].

For the plant’s ITC, i.e., MG, various studies have been carried out in both in vitro and in vivo models of NDDs with satisfactory results, as will be discussed hereunder.

MG as an antioxidant scavenged free radicals produced by iNOS, NO, and nitrotyrosine, and increased the nuclear level of the antioxidative marker Nrf2 in LPS-activated macrophages [92,177,279] and those of its downstream antioxidative genes; NQO-1, HO-1, GST-P1 [280]. As stated by Jaafaru et al. [281], GMG-ITC might stimulate cells to produce natural antioxidants at a low level, even in the absence of H₂O₂ damage. This was demonstrated by Giacoppo et al. [8], whereby MG was shown to suppress ROS formation in an oxidative stress environment to a minimum through its antioxidant characteristics. As indicated by flow cytometry-based analysis of ROS generation, pre-treatment of differentiated SH-SY5Y cells with 1.25μg/mL purified MG dramatically reduced ROS production and the expression of cyt-c, p53, Apaf-1, Bax, CASP3, CASP8, and CASP9 genes, while simultaneously increased the expression of Bcl-2. Consequently, Jaafaru et al. [281] hypothesized that pre-treatment with MG could mitigate oxidative stress in brain cells by lowering ROS levels and protecting cells from death via NDD probable pathways [281]. MG also lessened the expression of proinflammatory markers (TNF-α, IL-1β, and IL-6) in the brain, and that for P-selectin in the vascular endothelium of the brain and upregulated anti-inflammatory markers (IL-10 and TGF-β) [94,279,282]. MG also reduced the expression of the pro-inflammatory marker TLR-4, which prevented IκB-α degradation and NF-κB(p65) activation. Besides this, MG partially increased the expression of IκB-α, reduced Bax, and increased Bcl-2 [279]. Galuppo et al. [177,181,282] in a series of studies uncovered how MG modulates the NF-κB pathway: it suppressed expression and phosphorylation of ERK p42/44, Akt, and p38, thereby inhibiting IκB-α phosphorylation and activation and nuclear translocation of NF-κB. In the reduction of NF-κB and cytokine levels, MG was found to be more effective than SFN. Then, MG was considered to be more significant than SFN at inhibiting NF-κB activity [283]. Interestingly, a transcriptomic study showed MG treated human gingival mesenchymal stem cells (hGMSCs) suppressed the pro-inflammatory TNF-α, IL-1, IL-6 signaling, and upregulated the anti-inflammatory TGF-β as well as PI3K/AKT/mTOR pro-survival pathways [284]. MG showed its anti-inflammatory effect at a low dose (5 μM) [279].

In rat left carotid artery occlusion model of cerebral-ischemia reperfusion (CIR) injury, MG lowered MMP-9, iNOS and p-selectin expression, TNF-α level, and inflammatory cells recruitment within seven days after CIR induction. Furthermore, MG significantly reduced infarct volume, cerebral hemisphere edema (returning it to normal volume), functional disruption of neurons, and it prevented cerebellar detachment and resultant gait changes through modulating the phospho-ERK p42/44-mediated NF-κB pathway [181]. A post-investigation of EAE mice published recently firmly demonstrated that pharmacological treatment with GMG-ITC would stop the inflammatory cascade that underpins the mechanisms leading to acute multiple sclerosis [282]. The anti-inflammatory cytokine TNF-α and MAPK signal pathway were both well inhibited by MG. Furthermore, MG exhibited neuroprotective benefits against multiple sclerosis, reducing both clinical and histological symptoms associated with the condition (lymphocytic infiltration and demyelination) [282]. Further to that, Giacoppo et al. [91], uncovered that MG beneficially regulated the aberrant Wnt-β-catenin pathway resulting in GSK3β suppression and β-catenin up-regulation. MG also regulated T-cell activation and eliminated inflammatory mediators via the activation of PPARγ [91]. In another multiple sclerosis model, a study revealed the efficacy of a topical MG cream in lowering inflammation, clinical and histological disease scores, and enhancing remyelinization. Additionally, the MG cream was able to modify voltage-gated ion channels; alleviated neuropathic pain and encouraged hind limb recovery in the mouse model [182]. In MPTP experimental animal model of PD, MG protected neuronal cells from programmed cell death (downregulating STAT-1, p53, and p21 expression) and increased protein expression of TH, which may increase dopamine synthesis in the brain. Overall, MG abolished MPTP-induced neuronal and synaptic damage in substantia nigra, preserved normal behavior in pole test (for PD related bradykinesia), and improved the body weight of mice [92]. The exceptional influence of MG demonstrated in the study prompted the authors to suggest the need for the ITC to be explored further in clinical trials for treatment and/or prevention of PD [92].

Going further, pretreatment with MG protected against degeneration of spinal cord reticular fibers in spinal cord injury (SCI) mice and reduced severity of the disease due to suppressive effect on inflammation (increased IκB-α expression, prevented IκB-α degradation, and reduced NF-κB p65 expression) and apoptosis (reduced Bax and caspase 3 expression, increased Bcl2 expression) [93]. In the treatment of SCI, moringin-enriched liposomes in genetically modified stem cells (GMSCs) provided rapid recovery of motor function (hind limb motor function) after SCI by four days’ delay. MG improved disease scores and restored the morphology of the injured spinal cord to normal through modulating COX-2- and cytokine-mediated inflammation [94]. It also normalized glial fibrillary acidic protein (GFAP) expression and abolished expression of MMP9 and p38 [94]. Concerning amyotrophic lateral sclerosis (ALS), MG delayed development of motor deficits. The experiment showed improved conversion of T-helper to Treg cells, lowered CK enzyme level, decreased iNOS, TNF-α, TLR4, and CD8a, increased Nrf2 activity and reduced apoptosis (by reducing cleaved-caspase 3 activity and Poly (ADP-ribose) polymerase 1 (PARP-1) expression) [95].

However, on account of its poor solubility and stability in aqueous medium, the anti-inflammatory effect of MG was improved by complexation with α-cyclodextrin (α-CD) (MG/α-CD complex) [177]. A more promising effect was demonstrated in LPS-stimulated raw macrophages. MG/α-CD complex formed a stable inclusion system, which enhanced the water solubility and stability of MG [177]. In addition to this study, MG/α-CD complex has been tested for other bioactivities with good results [90,183]. A bioactive formulation of MG/α-CD complex was prepared by Mathiron et al. [285] using a combination of NMR and mass spectroscopy techniques. In a neuroblastoma model, the complex reduced cell growth by inducing apoptosis through activation of NF-κB/p65 and downregulation of MAPK and PI3K/Akt/mTOR pathways [183]. An in vitro (retinoic acid-differentiated SH-SY5Y cells exposed to Aβ_1–42_) transcriptomic study conducted with MG/α-CD complex on the treatment of AD showed suppression of disease progression and enhanced neuronal repair [90]. MG/α-CD reduced the expression of genes involved in senescence, autophagy, and mitophagy, all cellular processes that play a key role in the aggregation of Aβ and tau phosphorylation. Through downregulating the Slit/Robo signaling pathway, MG/α-CD induced neuronal remodeling and protected neurons against Aβ_1–42_ toxicity [90]. Cyclodextrins have been known for improving the physicochemical properties of various molecules. In a recent study, complexing hinokitiol with α-CD, β-CD, or γ-CD produced longer lasting antimicrobial activity and reduced minimum inhibitory concentration (MIC) than hinokitiol used alone against *Bacillus subtilis, Staphylococcus aureus, Escherichia coli, Pseudomonas aeruginosa, Candida albicans,* and *Aspergillus brasiliensis* [286].

Surprisingly, though MG preserved the viability of normal neuronal cells (as well as microglia and astrocytes) and also produced anti-apoptotic effect as discussed in the preceding paragraphs, it was able to cause cytotoxic effect—at high concentrations: (16.4 µM and 24 μM) in CCF-STTG1 (human grade IV astrocytoma) cells and SH-SY5Y cells [96,287]. This property has been experimented on human malignant astrocytoma cells (CCF-STTG1), where MG induced apoptosis via inciting oxidative stress in CCF-STTG1 cells with decreased expression of p53, CKIIα (casein kinase II), and Nrf2 levels. Through this mechanism and partly through ribosome assembly perturbation, MG caused morphological changes in CCF-STTG1 cells, including deeply stained nuclei, condensed cytoplasm, and cell death, with severe cell death at >/= 32 μM [96]. Apart from inducing apoptosis, MG reduced cell growth and caused accumulation of SH-SY5Y cells at G2 phase, thereby reducing tumor burden by up to 90% at 16.4 µM when administered for 72 h. It increased Bax, p53, p21 expression and reduced Bcl2 expression, as well as increased gene expression and cleavage of caspase 3 and 9 [287]. Findings by Jaafaru et al. [36] clearly demonstrated the safety of GMG-ITC-RSE (glucomoringin-isothiocyanate-rich soluble extract) in vivo (acute toxicity test on Sprague Dawley rats) and in vitro, while determining its anti-proliferative action on human prostate adenocarcinoma cells (PC-3) cells. The use of GMG-ITC-RSE had no detrimental effects on the rats, even at large doses (2000 mg/kg body weight), and no untoward effect on any of their important organs [36]. In 3T3 preadipocyte cells, the extract showed great level of safety with more than 90% of the cells remaining viable after treatment with the extract in a time-dependent manner, even at high doses (250 g/mL). Under the microscope, GMG-ITC-RSE strongly caused morphological abnormalities that are characteristic of apoptosis [36]. As stated by Brunelli et al. [22], both SFN and MG were able to induce cell death in the micromolar range in all tested cell lines, but caused cell cycle perturbations only in myeloma cells and they were also able to modulate the GST/GSH pathway by causing a three-fold increase in GST-π activity in human breast cancer cell line (MCF7) cells. In this investigation, the purified MG demonstrated a strong antitumoral effect in a myeloma mouse model with little toxicity [22]. Thus, the authors concluded that MG appears to be a promising choice for further research into its potential in myeloma treatment, having shown that MG efficiently inhibited NF-κB activity and induced apoptosis through a caspase-dependent pathway [22]. Researchers have stressed on the importance of the ITC group of MG in producing chemopreventive effects. Cytotoxic effect tends to reduce as MG degrades in alkaline and acidic/neutral pH by isomerization or hydrolysis during food processing [288].

Taken together, MG displayed interesting features in modulating oxidative stress, inflammation and apoptosis, which are central to the development and progression of NDDs. The resultant effects made it possible for MG to reduce infarct volume in ischemic stroke [181], suppress demyelination in multiple sclerosis [91], increase expression of TH in PD [92], delay onset of disease manifestation in ALS [95], reduce severity of SCI [93,94], among other protective effects. It is worth commenting that the setback of MG on solubility and stability has been improved by the development of a more soluble and stable bioactive formulation, courtesy of Mathiron et al. [285]. This complex presented greater positive effects in models tested so far as discussed above. The parent plant, *M. oleifera,* showed neuroprotective effects that helped preserve viability of neurons and protected them from cytotoxicity in AD [278] and focal stroke model [88].

#### 5.2.4. Erucin (ER)

Neuroprotective effect is currently demonstrated by the ability of a substance to provide anti-oxidative, anti-inflammatory, and/or antiapoptotic effects [16,60]. In NSC-34 motor neurons exposed to the culture medium of LPS-stimulated macrophages, *E. sativa* (*Eruca sativa*, Mill.) extract (ESE)—containing high amount of ER—prevented LPS-induced cell death and degeneration by counteracting apoptosis (inhibit FasL (tumor necrosis factor ligand superfamily member 6) expression), suppressing pro-inflammatory mediators (attenuates COX-2 and TLR4 expression and TNF-α level) and stimulating release of the anti-inflammatory cytokine IL-10 [98]. ESE abolished the expression of NLRP3 inflammasome and consequently the activation of caspase 1 and production of IL-18 and IL-1β [98]. In an in vitro dopaminergic-like neuroblastoma cell line model (6-OHDA-induced SH-SY5Y) of PD, ER increased resistance of DAergic neurons to apoptosis through upregulating GSH antioxidant activity. ER treatment at 2.5 and 5 μmol/L for 24 h resulted in increased intracellular GSH content by about 35 and 45%, respectively, and overall total antioxidant capacity (TAC) in the cytosol [289]. ER also prevented disruption of mitochondrial membrane potential, redox status impairment, and intracellular ROS formation, especially that of O_2_^-^ [289]. Still on PD, ER produced similar protection to SFN against experimental 6-OHDA-induced PD especially in the in vivo model (6-OHDA-PD mouse model). ER demonstrated Nrf2-induced activation of GSH synthesis thereby improved TH secretion in the substantia nigra and suppressed DAergic neuron apoptosis [103].

From the little research of ER on PD, it is possible to say that this ITC may improve dopamine levels in the brain [103]. More research is needed to establish how and to what extent this occurs.

#### 5.2.5. Allyl Isothiocyanate (AITC)

AITC is generated from sinigrin hydrolysis, a GL common in Brassica crops; *Wasabia japonica* (wasabi) is rich in AITC [104]. Experimental findings revealed that the neuroprotective effect of AITC against LPS-induced neuroinflammation is mediated through downregulation of JNK/NF-κB/TNF-α signaling, while neuronal death is mediated by downregulation of mitochondrial apoptotic pathway in neuroblastoma cells [290]. In an experimental model of traumatic brain injury, AITC reduced brain infarct volume and to some extent the brain swelling ensuing extravasation effect of inflammation [107]. The ability of AITC to increase the expression of growth-associated protein 43 (GAP43) and neural cell adhesion molecule (NCAM) in the brain tissue proclaimed that AITC may promote regeneration of neurons after injury. Also, AITC inhibited the activation of GFAP responsible for astrogliosis as well as preserved BBB integrity [107]. In a study that compared the effects of SFN, PEITC, and AITC on neuroinflammation, all ITCs were found to decrease the expression of MMP-2 and MMP-9, though only SFN significantly reduced circulating MMP-2 level via downregulation of ERK phosphorylation [291]. Interestingly, the effects of AITC upon binding to bitter taste receptor (TAS2R) were shown to be individual specific. Due to differences in functionality of the receptor, healthy human volunteers responded differently to AITC and only those with functional receptor haplotype experienced beneficial effects of AITC [292]. NO-dependent bactericidal activity, inhibition of TNF-α secretion, and MAPK and PI3K/AKT-dependent anti-inflammatory activity by AITC both depended on TAS2R38 genotype receptor. Though without significant differences in AITC plasma levels in volunteers, AITC produced lesser effects in TAS2R38 negative volunteers [292]. Recently, AITC complexed with β-cyclodextrin and carbon nanotubes was used to study its effect on microbes; the complex maintained slow release of the antimicrobial agent [293]. This strategy can be employed for the assessment of other effects of AITC and related compounds.

#### 5.2.6. Indole-3-Carbinol (I3C)

In neuronal cells, I3C suppressed free radical production [294] and chelated already produced radical species through potent radical scavenging activity [295]. DIM inhibited NF-κB activity and IκB phosphorylation, thereby suppressing expression of iNOS, NO, and COX-2 in the brain, which averted inflammation and apoptosis [296]. In mice pretreated with DIM before LPS injection, marked suppression of hippocampal inflammation was observed in comparison to vehicle-treated disease group [296,297]. Also, DIM counteracted oxidative stress and apoptosis in hippocampal cells as a result of tropomyosin-related kinase receptor B (TrkB)/Akt pathway activation. The activation promoted synthesis of brain-derived neurotrophic factor (BDNF) and antioxidant mediators (HO-1, NQO-1, and GCL-C) [112]. Due to preservation of hippocampal neuronal cells, enhancement of ChAT activity and reduction of AChE activity, I3C averted memory impairment in scopolamine-treated mice [112]. Another derivative of I3C, i.e., [1(4-chloro-3-nitrobenzenesulfonyl)-1H-indol-3-yl]-methanol (CIM), suppressed P/Q-type Ca^2+^ channels and Ca^2+^/calmodulin/adenylate cyclase/cAMP/protein kinase A pathway, which caused inhibition of vesicular glutamate transporter and glutamate release from synaptosomes [298]. This may be beneficial in alleviating excitotoxicity in NDDs [298].

I3C may delay neurodegeneration and improve motor symptoms, coordination, learning, and memory in PD by suppressing NF-κB-induced inflammation. Apart from marked suppression of neuroinflammation (decreased TNF-α and IL-6) and preservation of dopaminergic neurons in the substantia nigra, I3C also reduced lipid peroxidation in the cortex far greater than levodopa-carbidopa combination [113]. As the synergistic effect achieved with combination of I3C with levodopa-carbidopa enhanced resolution of oxidative stress and inflammation, the authors suggested addition of I3C to levodopa-carbidopa in the treatment of PD [113]. In AD, I3C lessened Aβ_1–42_ aggregation and at high concentration inhibited Aβ_1–42_ fibril polymerization [114]. Comparable but superior effect has been achieved on Aβ_(25–35)_, as I3C significantly inhibited and destabilized already formed Aβ_(25–35)_ aggregates and prevented Aβ_(25–35)_-induced damage to brain mitochondria [299]. Moreover, I3C’s induction of AhR promoted endogenous Aβ catabolic enzyme Neprilysin expression and activity, reduced Aβ_42_ levels and ameliorated cognition dysfunction; it was proposed that AhR might serve as a therapeutic target in the treatment of AD [115]. In a very similar manner to SFN [263], I3C and DIM reduced severity of EAE and absolutely suppressed clinical features and T-cell infiltration in the CNS [116]. I3C modified T-cell responses with decreased Th17 and increased Treg cell and FoxP3 formation by acting through AhR [116]. Anti-thrombotic and antioxidant effects of I3C may make it a potential candidate for prophylactic and therapeutic use in cerebral ischemic stroke. In MCAO rat model, I3C inhibited platelet aggregation, reduced infarct volume, improved mean cerebral blood flow, and improved neurological scores [117].

On the other hand, I3C and its derivatives (DIM and others) showed varying degrees of potency on resistant glioblastoma cell lines and short-term glioblastoma cultures [300]. I3C inhibited NEDD4-1 (neuronal precursor cell-expressed developmentally downregulated 4–1)—which physiologically regulates embryonic development—with downstream suppression of both tumor suppressor PTEN (phosphatase and tensin) homolog ubiquitination and Nrf2/HO-1 signaling pathway activation. This novel anticancer effect of I3C promoted sensitization of temozolomide-resistant glioblastoma cells both in vivo (resistant glioblastoma cells inoculated female nonobese diabetic/severe combined immune-deficient (NOD/SCID) mice) and in vitro (temozolomide-resistant glioblastoma cell line) to the cytotoxic effect of the anticancer drug [118]. I3C has poor BBB penetration, therefore I3C-loaded nanoparticles (NPs) with poly(D,L-lactic-co-glycolic acid) (PLGA) stabilized by Tween 80 (T80) (I3C-PLGA-T80-NPs) were formulated, which easily crossed the neuronal cytoplasmic membrane of PC12 neuronal cells [301]. The efficacy of the formulation was tested on PC12 neuronal cells injured with glutamate excitotoxicity where it blocked ROS production [301].

Together, these findings have explained the ability of I3C to attack oxidative stress in the brain [294,295], dampen neuroinflammation [296,297], arrest neuronal apoptosis, as well as stimulate neuronal regeneration [112]. Regularization of the cholinergic system by I3C helped memory improvement [112], while marked inhibition of excitotoxicity may improve pathogenic mechanisms in various NDDs [298]. I3C rescued dopaminergic neurons from injury in PD [113], while in AD it stopped aggregation and polymerization of Aβ fibrils [114] and destroyed already formed ones [299]. Subsequently, AhR has been proposed as a novel target in AD treatment [115]. Many beneficial effects were demonstrated in multiple sclerosis [116] and ischemic stroke [117] models. Not surprisingly (i.e., similar to other ITCs), I3C presented chemopreventive effect; increased temozolomide sensitivity in otherwise resistant tumor cells [118].

Table 3 below presents the beneficial effects and mechanisms of action of ITCs (SFN, PEITC, *M. oleifera* Lam. (Moringaceae)/MG, ER, AITC, I3C) on various models of NDDs.

## 6. Adverse Effects of Glucosinolates (GLs) and Isothiocyanates (ITCs)

Accumulating evidence over the preceding three decades has shown the toxicity profile of GLs and ITCs [302]. Black cabbage seed extract containing appreciable quantity of GRA caused generalized suppression of phase I metabolizing enzymes in rat liver and lungs and it was documented that high dose use may drastically hinder the metabolism of endogenous substances that are substrates of cytochrome enzymes [303]. On the other hand, GRA has been shown to cause DNA damage and powerful induction of cytochrome P450 enzymes, which aside from chemopreventive effect, also caused bioactivation of polycyclic aromatic hydrocarbons (PAH) and escalated PAH conversion to carcinogenic form in rats. Therefore, regular administration may increase the risk of cancers in individuals exposed to environmental mutagens and/or carcinogens [304]. GL-rich (37.3 umol/g of dry matter) diet in rats caused reduction in feeding and growth rate, with about 36% mortality. Demonstrable enlargement of the thyroid gland, liver, and kidney, as well as reduced plasma thyroid hormone levels were recorded [305]. Other hydrolytic products of GLs, especially oxazolidine-2- thiones (epigoitrin, progoitrin, goitrin and glucoconringin) and thiocyanates, may also suppress thyroid hormone synthesis [24,26]. High content of goitrogens (GMG, cyanogenic glucosides, thiocyanate, and polyphenols) in moringa (*M. oleifera* Lam.) plant also caused biochemical and morphological hypothyroidism in male albino rats [306].

Gastrointestinal tract irritation, abdominal pain [307], and leucopenia have been reported for AITC, while SFN (64 mg/kg) led to hepatotoxicity [10]. In the stomach milieu, I3C may spontaneously condense to form compounds that resemble the toxin named dioxin (2,3,7,8-tetrachlorodibenzo-p-dioxin, TCDD) in both structure, toxicity, and carcinogenicity profile [26]. Also, nitriles and progoitrin have been linked with anemia and pancreatic damage, respectively [307]. Different pathways may be at play with regards to toxicity of GLs and ITCs; on one hand responsible for thyroid dysfunction and on the other for phase I enzyme induction [305]. Although concentrations that give rise to toxic effects (in mutagenic studies for example) are so much higher than concentrations achieved after regular dietary intake, it is still paramount to determine the therapeutic window of ITCs [10]. Toxicity studies for *M. oleifera* showed safety levels up to 3000 mg/kg [308]. Interestingly, in postmenopausal and female diabetic volunteers that were given different forms of moringa leaf powder, no adverse effects were reported [308]. Therefore, these toxicities may not completely restrict their use for improving health and wellbeing.

Breeding programs centered towards reducing toxicity of GLs including breeding crops with low GLs content have been implemented for some Brassica species and are underway for others [24]. At the same time, to improve the benefits derived from these vegetables, GLs biofortification targeted at breeding crops to selectively increase beneficial GLs and agronomic biofortification, which may alter GLs’ concentration based on environmental conditions before and after harvest are also being considered [24].

## 7. Conclusions

Numerous research publications have established the biological properties of phytochemicals and especially in relation to critical diseases of humans. The beneficial biological properties of phytochemicals from Brassica vegetables, such as GLs, have lured many researchers’ interest towards the development of effective treatment, for both metabolic and non-metabolic diseases in relation to cardio- and neuroprotective effects. The significant effects of GLs stand as a result of their conversion by myrosinase to diverse ITC compounds. Yet, the most studied ITC is SFN, which is found in the Cruciferae family in the form of GRA, its precursor GL. SFN has been demonstrated in several in vitro and in vivo models and in clinical investigations to potentially contribute to the prevention of both CVDs and NDDs.

Besides, the aforementioned ITCs are extremely effective protective agents because of their capacity to produce varied and long-lasting responses that defend against oxidative stress, electrophilic stress, chronic inflammation, and apoptosis. However, the lack of a sufficient GLs food composition database, which takes into account food processing-induced changes, makes it particularly problematic to estimate GLs daily dietary consumption. Therefore, in order to conduct more chemoprotection studies, it will be important to fully know and manage the parameters that influence the toxicity dose of GL and ITC conversion, as well as genotoxicity in humans. However, processes are under way to develop a GLs database in some countries like the US, but lack of adequate information among other reasons have hindered going further [309].

## Figures and Tables

**Table 1:** Isothiocyanates major food sources and main effects on cardiovascular diseases (CVDs) and neurodegenerative diseases (NDDs).

Phytochemical	Major Food Sources	Main Effects on CVDs/Risk Factors	Main Effects on NDDs
SFN	Broccoli, cauliflower, kale, brussels sprouts, cabbage [15,16]	Reduced obesity, normalized serum lipids, increased plasma insulin, decreased blood pressure, slowed progression of atherosclerosis, and prevented vascular complications in diabetes mellitus (DM) [61] Suppressed myocardial damage, decreased infarct area in myocardial infarction (MI)[62] Improved cardiac function in arrhythmia, MI, and heart failure [62,63,64] Regularized heart rate in arrhythmia [64] Reduced severity of right heart failure/pulmonary hypertension [65] Decreased heart muscle dysfunction in the elderly [66]	Reduced cholinergic neurons’ apoptosis, improved cholinergic neurotransmission, and neurobehavioral responses [67,68] Downregulated amyloidogenesis [68,69] Inhibited dopaminergic neuron death, increased tyrosine hydroxylase (TH) formation in Parkinson’s disease (PD) [70,71] Decreased brain infarct in ischemic stroke and neonatal hypoxia-ischemia injury [72,73] Prevented motor neuron death, decreased severity and incidence of multiple sclerosis (MS), epilepsy and, depression [74,75,76] Reduced side effects of schizophrenia medications [77]
PEITC	Turnips, radish, watercress, broccoli [78]	Reduced food intake, body weight, fat deposition, and atherosclerosis [79,80] Normalized plasma insulin and blood glucose [81] Suppressed left ventricular dysfunction in HIV/AIDS [82]	Inhibited acetylcholinesterase activity [39]
MG	Moringa seeds and leaves [22]	Minimized MI size, decreased creatine kinase MB (CK-MB), improved cardiac function, and reduced mortality after MI [83,84] Reduced disease severity in heart failure [85] Decreased fat accumulation, increased lean body mass, improved blood glucose, and gut microbiome in DM [86]	Promoted neurogenesis and viability [87] Improved neurological score and decreased infarct size in stroke [88] Improved neurocognition, normalized catecholamines and electroencephalogram (EEG) wave pattern, suppressed disease progression, and enhanced neuronal repair in AD [89,90] Decreased demyelination and improved remyelination in MS [91] Increased TH production, decreased dopaminergic neuron apoptosis, improved behavior and motor symptoms in PD [92] Suppressed progression of spinal cord injury and hastened motor function recovery [93,94] Delayed development of motor deficits in amyotrophic lateral sclerosis (ALS) [95] Induced apoptosis in astrocytoma and myeloma [22,96]
ER	Arugula, kohlrabi, Chinese cabbage kohlrabi, broccoli seeds [97,98]	Reduced body mass index, lipid accumulation, serum triglycerides, fasting blood glucose, hemoglobin A1C [99] Decreased blood pressure [100,101] Suppressed platelet aggregation and thrombosis, improved coronary blood flow [100] Decreased CK-MB and lactate dehydrogenase (LDH) [102]	Increased resistance of dopaminergic neurons to apoptosis and increased TH secretion in PD [103]
AITC	Wasabi, mustard, horse radish [104]	Suppressed insulin resistance, decreased blood glucose, reduced obesity, decreased cholesterol synthesis, reduced MABP [105,106]	Decreased infarct volume from traumatic brain injury [107]
I3C	Broccoli, brussels sprouts, cabbage [15,16]	Anti-platelet, anti-thrombotic activity [108] Prevented myocardial hypertrophy, stimulated parasympathetic effect [109] Normalized cardiac nitric oxide, decreased CK-MB [109] Increased insulin, decreased blood glucose, HbA1C, and cholesterol [110] Suppressed lipid deposition in blood vessels [111]	Improved cholinergic neurotransmission and memory [112] Decreased dopaminergic neuron loss [113] Ameliorated cognitive dysfunction, inhibited formation and aggregation, and also promoted degradation of amyloid beta plaques [114,115] Decreased severity of MS [116] Increased neurological score and cerebral blood flow, and decreased infarct volume [117] Increased sensitivity of temozolomide-resistant glioblastoma cells [118]

**Table 2:** The beneficial effects of ITCs (SFN, *M. oleifera, E. sativa*/ER, I3C) on CVDs with proposed underlying mechanisms of action.

ITC or EAxtract	CVD and/or Model	Effect on CVDs	Mechanism of Action	References
SFN	Myocardial infarction (MI)/surgical left coronary artery occlusion in rats	Decreased heart congestion and remodeling	Upregulated MAPK/Akt/ERK pathway and downregulated p38 and Bax/Bcl-2-caspase-3 pathways	[135]
		Preserved cardiac function and reduced infarct size more than postC	Balanced Nrf2/AhR activation	[62]
	MI/Hypoxia/reoxygenation (H/R) myocardial cells model	Restored cardiac anti-oxidant status, reduced apoptosis	Activated Nrf2/HO-1 pathway	[152]
	CVD/Mutated GATA cardiomyocytes (in vitro)/isoproterenol-induced cardiac hypertrophy in mice (in vivo)	Suppressed cardiac hypertrophy	Inhibited GATA4/GATA6 expression and MAPK signaling pathway	[153]
	Chronic heart failure (CHF)/Doxorubicin (DOX)-induced CHF	Retarded disease progression and improved heart function	Stimulate Nrf2 transcription, inhibited PAI-1 and CTGF expression	[73]
	Arrhythmia/Isoproterenol-induced cardiac stress in rat	Normalized heart rate and improved left ventricular function	Normalized cardiac autonomic drive	[64]
	Pulmonary arterial hypertension (PAH)/VEGFR inhibitor (SU5416)-induced PAH in mice	Prevented right ventricular and pulmonary vascular dysfunction and remodeling	Reduced NLRP3 expression and upregulated Nrf2/NQO-1 pathway	[65]
	Cardiomyopathy (CM)/Angiotensin II induced cardiomyopathy in mice	Suppressed cardiac oxidative stress, inflammation, remodeling, and dysfunction	Epigenetic modification of Nrf2 activation with HDAC and DNMT inhibition	[157]
	Diabetic CM/Type I DM OVE 26 (OVE) mice	Improved cardiac function and ameliorated fibrosis	Increased Nrf2 activity and metallothionein expression	[158]
	CM/Aged-mice cardiac muscle dysfunction	Improved cardiac and mitochondrial function	Upregulated Nrf2 signaling	[66]
	Chromium heart toxicity/Chromium (CrVI)-induced cardiotoxicity	Ameliorated cardiac physiological and morphological alterations	Activated Sesn2/AMPK/Nrf2 signaling pathway	[159]
	Diabetic vascular injury/AGEs-exposed HUVECs and AGEs-injected rat aorta	Antioxidative, anti-inflammatory	Inhibited AGE/RAGE pathway	[61]
	CVD/Rat aortic smooth muscle cells (RASMCs)- in vitro; rat carotid artery balloon injury model – in vivo	Inhibited neointima formation	Inhibited PDGF-BB-stimulated proliferation of RASMCs, by causing cell cycle arrest through downregulating the p53 signaling pathway	[149]
	CVD/H_2_O_2_-exposed adult cardiomyocytes	Antioxidative: reduced ROS and raised SOD	Induced Nrf2 and PGC-1α protein expression	[133]
	CHF/aortic constriction in rabbits	Improved heart function and remodeling	Inhibited oxidative stress and inflammation (↓TNF-α, ↓IL-6) and decreased BNP and ANP	[63]
*M. oleifera* extract	MI/left coronary artery ligation in mice	Minimized infarct sizes, alleviated contractile dysfunction, prevented ventricular failure, and reduced mortality	Repressed oxidative/nitrosative stress, apoptosis, and fibrosis	[83]
	CHF/DOX-induced CHF	Reduced serum LDH, CK-MB, normalized ECG parameters, and reduced mortality	Increased cardiotonicity	[85]
	MI/isoproterenol-induced myocardial damage in rats	Improves cardiac performance, antioxidative, antiperoxidative, and myocardial preservative effects	Restores hemodynamic parameters, prevents leakage of LDH and CK-MB from the myocardium, SOD, CAT, and GSHPx	[84]
ER	Hypertension/HASMCs, noradrenaline-induced vasoconstriction endothelium-intact or -denuded rat aortic rings, coronary arteries of Langendorff-perfused rat hearts and normotensive and SHRs	Vasorelaxant, antihypertensive effect	H2S-releasing	[100]
*E. sativa*	Hydroxyapatite cardiac toxicity/Hydroxyapatite-induced cardiac damage	Lowered CK-MB, LDH, and myoglobin	-	[102]
I3C	CVD/DOX-induced cardiotoxicity	Raised cardiac antioxidant status Reduced oxidative stress, inflammation, and apoptosis	-	[218]
			Upregulated Nrf2/ARE pathway, downregulated NF-kB pathway, modified apoptotic genes’ expression	[220]
	Heart failure/Aortic banding in mice	Prevented pressure overload-induced cardiac remodeling	Activated AMPK-α signaling and improved energy metabolism	[108,219]
	Hypertension/High salt-induced myocardial stress and hypertrophy	Anti-hypertensive, anti-hypertrophic, and anti-apoptotic effects	Stimulation of muscarinic receptor-2	[109]

**Table 3:** Beneficial effects and mechanisms of action of ITCs (SFN, PEITC, *M. oleifera* Lam. (Moringaceae)/MG, ER, AITC, I3C) on various models of NDDs.

Isothiocyanate (ITC) or Extract	Neurodegenerative Diseases (NDD) and/or Model	Effect on NDDs	Mechanism of Action	References
SFN	Alzheimer’s disease (AD)-like mouse model	Abolished apoptosis of cholinergic neurons, reduced cognitive impairment	Probably neurogenesis and aluminum load reduction	[239]
	Amyloid bete (Aβ)-induced AD acute mouse model	Improved cognitive function	-	[238]
	Transgenic AD mouse model	Ameliorated neurobehavioral deficits and reduced Aβ burden	Increased expression of p75NTR	[67]
	D-galactose and aluminum-induced AD-lesion mouse model	Improved cognitive and locomotor function	Suppressed Aβ deposition	[68]
	Scopolamine-induced memory impairment in C57BL/6 mice (in vivo), scopolamine-treated primary cortical neurons (in vitro)	Improved cholinergic neurotransmission, memory, and cognition.	Inhibited acetylcholinesterse (AChE) activity	[241]
	AD/ Aβ-induced-SH-SY5Y cells	Antiapoptotic	Stimulated Nrf2 pathway	[242]
	AD transgenic mouse (PS1V97L)	Improved spatial learning and memory	Inhibited Aβ oligomer formation	[69]
	Triple transgenic AD mouse model (3×Tg-AD)	-	Enhanced Aβ and tau degradation via increased CHIP and HSP70 expression	[243]
	AD/Mouse neuroblastoma N2a cells expressing human Swedish mutant amyloid precursor protein (N2a/APPswe cells)	Inhibited oxidative and inflammatory effects of Aβ	Epigenetic modification of Nrf2	[244]
	Aβ_1–42_ induced-human THP-1 macrophages (in vitro AD model)	Suppressed neuroinflammation	Preserved MerTK expression via NF-κB pathway downregulation	[245]
	AD/ Aβ_1–42_ monomers induced human THP-1 microglia-like cells	Anti-inflammatory effect (decreased IL-1β)	Inhibited activation of STAT-1 and NLRP3 inflammasome, decreased microRNA-146a and upregulated Nrf2 pathway	[246]
	AD/Aβ oligomer-induced microglial cells	Anti-inflammatory effect	Improved microglial phagocytic activity	[247]
	AD/Aβ_1–42_-induced cytotoxicity in Neuro2A and N1E115 cells	Anti-oxidant effect	Increased proteasome (PSMB5) Aβ degradation	[248]
	NDD/MG132-induced proteasome inhibition in Balb/c mice	Improved spatial learning	Induced catalytic activity of proteasome	[249]
	Parkinson’s disease (PD)/MPTP-induced sub-acute model	Prevented dopaminergic neuron loss, micro- and astrogliosis	Upregulated Nrf2 mediated phase II enzymes expression	[254]
	PD/6-OHDA- induced PC12 cells	Anti-apoptotic	Enhanced PI3K/Akt-dependent HO-1 expression	[213]
	PD/6-OHDA- induced ER stress in PC12 cells	Anti-oxidative	Improved Nrf2 inhibition of endoplasmic reticulum (ER) stress	[253]
	PD/ 6-OHDA- and BH4- induced SK-N- BE(2)C, CATH.a and mesencephalic neurons	Prevented dopaminergic cell death	Removal of dopamine quinone from neuronal cells	[251]
	ALS/Threo-hydroxyaspartate (THA)-induced glutamate excitotoxicity on spinal cord explant model	Decreased motor neuron death	Induction of phase II enzymes via Nrf2/ARE signaling	[261]
	PD/CysDA-induced primary cortical neurons injury	Abolished oxidative stress and apoptosis	Upregulated ERK/Keap1/Nrf2 pathway	[70]
	PD/6-OHDA-induced mouse model	Improved behavior and motor coordination	Downregulated phosphorylation of ERK1/2, increased GSH and GR, and blocked expression of caspase-3	[71]
	PD/H_2_O_2_ or 6-OHDA-induced cytotoxicity in SH-SY5Y cells	Anti-apoptotic	Induced GSH-mediated antioxidative response	[250]
	PD/Acute and sub-acute MPTP models in C57BL/6 mice	Improved behavior, coordination, and motor function	Reduced dopamine transporter degradation, increased tyrosine hydroxylase (TH) expression. Normalized expression of neurotrophic factors; GAP-43, NGF, and BDNF	[255]
	* Stroke/rat common carotid/middle cerebral artery (CCA/MCA) occlusion model	Reduced infarct volume	Increased HO-1 expression	[72]
	* Stroke/carotid artery occlusion CIR injury in rats	Reduced infarct volume, restored BBB integrity	Decreased ERK1/2, NF-kB, and casp3 expression, increased Nrf2 activity	[256,258]
	* Stroke/rat MCAO model	Improved neurological scores and minimized infarct volume	Inhibited NLRP3 inflammasome and caspase-1 activation	[257]
	Perinatal hypoxia-ischemia/Neonatal HI rat model (left common carotid artery ligation and hypoxia)	Reduced infarct ratio	Induction of phase II enzymes through Nrf2 signaling	[73]
	* Stroke/bilateral common carotid artery occlusion (BCCAO) injury in rat	Lowered extent of acute cerebral injury	-	[260]
	NDD/oxygen and glucose deprivation OGD in rat cortical astrocytes	Suppressed astrocyte death	Stimulated Nrf2 pathway	[259]
	Multiple sclerosis (MS)/MOG35-55-induced EAE in C57BL/6 mice	Inhibited disease development and severity, suppressed spinal cord demyelination	Inhibited Th17 autoimmune response, upregulated Nrf2 pathway	[263]
	MS/MOG35-55-induced EAE mouse model	Improved BBB integrity	Increased expression of TJ-proteins, decreased Foxp3, ERK1/2 and caspase 3 expression	[264]
		Suppressed symptoms	Modulated inflammatory pathways, reduced apoptosis	[265]
	Prion diseases/PrP exposed-SH-SY5Y cells	Antiapoptotic	Induced autophagy by stimulating AMPK pathway	[266]
	Schizophrenia/anti-psychotics-induced SK-N-SH cells	Suppressed dopaminergic neuron toxicity	Decreased protein-bound quinones	[77]
	Epilepsy/Amygdala chronic kindling model	Suppressed amygdala kindling and cognitive impairment	Activation of Nrf2-ARE signal pathway	[75]
	Depression/Acute and chronic stress mouse model	Antidepressant- and anxiolytic-like activities	Inhibited HPA axis activity	[76]
PEITC	Spinal cord injury (SCI)/Dorsal column/Sciatic nerve injury in rats	Promoted neurite outgrowth	Modulated miR-17-5p/STAT3/GAP-43	[267]
*M. oleifera* extract	NDD/Primary culture of hippocampal neurons	Promoted neuronal survival and neurite outgrowth	-	[87]
	NDD/Al-induced temporo-cortical degeneration in mice	Reduced degenerative features	Increased NSE, decreased GFAP	[276]
	* Stroke/right MCAO model in rat	Improved clinical score, reduced infarct volume	Decreased MDA levels, increased SOD and GSHPx activity	[88]
	NDD/Hippocampal neurodegeneration rat model	Enhanced memory and cognition	Inhibited AChE activity	[277]
	AD/Colchicine-induced AD model	Improvement of RAM task and EEG wave pattern, normalization of serotonin, norepinephrine, and dopamine	-	[89]
	AD/Scopolamine-induced spatial memory deficit in mice	Improved spatial memory function	Maintained cholinergic transmission and neuron integrity	[278]
MG	MS/MOG35-55-induced EAE in C57BL/6 mice	Stopped TNF-α inflammation	Inhibited phospho-ERK p42/44 signaling pathway	[282]
		Decreased clinical disease score and inflammatory markers	Modulated Wnt–β-catenin signaling	[91]
	AD/Aβ-induced-SH-SY5Y cells	Slowed disease progression, promoted neuronal repair	Downregulated pathways involved in senescence, autophagy, and mitophagy	[90]
	PD/MPTP-induced sub-acute PD mouse model	Reduced bradykinesia	Suppressed inflammatory response, increased TH expression	[92]
	* Stroke/left carotid artery occlusion model in rat	Reduced infarct size, improved neurologic symptoms	Downregulated NF-κB pathway	[181]
	SCI/extradural spinal cord compression in ICR (CD-1) mice	Restored motor function, spinal cord morphology, and promoted regenerative effects	Increased expression of TGF-β and IL-10	[94]
		Reduced disease severity, prevented secondary spinal cord damage after injury	Downregulated NF-κB pathway	[93]
	Amyotrophic lateral sclerosis (ALS)/ALS transgenic model (SOD1^G93A^ rat)	Delayed appearance of motor dysfunction	Downregulated TLR4 and CD8α mediated inflammation, oxidative stress, and apoptosis	[95]
	Neuroblastoma (NBL)/ SH-SY5Y human NBL cell line	Stimulates cancer cell apoptosis	Inhibited PI3K/Akt/mTOR pathway	[183]
ER	PD/6-OHDA induced SH-SY5Y cells’ model	Antioxidative, antiapoptotic effects	Increased GSH level, prevented loss of mitochondrial membrane potential	[103,289].
	PD/6-OHDA induced mouse model	Counteracted asymmetric motor behavior	Increased TH expression	[103]
AITC	NDD/LPS-induced neuroinflammation model (BV2 murine microglia, C6 glioma, and N2a mouse neuroblastoma cells)	Antiapoptotic, improved neurite outgrowth	Suppressed JNK/NF-κB/TNF-α signaling	[290]
	Traumatic brain injury (TBI)/Cryogenic TBI model in mice	Improved infarct volume and BBB permeability	Modulated Nrf2/HO-1 and NF-κB pathways	[107]
AITC, PEITC and SFN	NDD/LPS-activated primary cultures of rat astrocytes	Anti-inflammatory	Modulated MMP transcription via downregulating MAPK/ERK signaling	[291]
I3C/DIM	NDD/LPS-induced microglial hyperactivation in BV-2 Microglia (in vitro)/mice (in vivo)	Suppressed neuroinflammation and apoptosis Reduced activated microglial cells in the hippocampus	Inhibited NF-κB	[296]
	NDD/glutamate-treated HT-22 Cells/Scopolamine-induced memory impairment in mice	Anti-apoptotic Improved cognition; reduced AChE activity and enhanced choline acetyltransferase (ChAT) activity	Activated TrkB/Akt pathway (increased BDNF and antioxidants)	[112]
	NDD/ 4-aminopyridine-treated synaptosomes	Inhibited glutamate release from nerve terminals	Downregulated Ca^2+^/calmodulin/protein kinase A pathway and P/Q-type Ca^2+^ channels	[298]
	NDD/glutamate excitotoxicity (GE) in PC12 neuronal cells	Anti-apoptotic	Scavenged ROS, inhibit caspase-3 and -8	[301]
	PD/intranigral LPS-induced neuroinflammation in rats	Improved motor functions, coordination, learning, and memory	NF-κB pathway inhibition	[113]
	AD/Aβ-induced PC12 cells	Inhibition of amyloid fibril formation, aggregation, and cytotoxicity	-	[114]
	AD/Aβ_(25–35)_-induced rat brain mitochondria	Inhibited amyloid fibrils formation, destroys amyloid aggregates	Inhibited mitochondrial membranes damage	[299]
	AD/Small interfering RNA knockdown and plasmid transfection model (in vitro)/ Adeno-associated virus-mediated RNAi in mice (in vivo)	Improved cognition and Aβ catabolism	Stimulated AhR-induced Neprilysin expression	[115]
	MS/MOG35-55-induced EAE in C57BL/6 mice	Reduced disease severity and T-cell infiltration in the CNS	Increased Treg cell/FoxP3 formation and decreased Th17 by activating AhR	[116]
	* Stroke/MCAO in rat	Improved neurological score and mean cerebral blood flow. Reduced platelet aggregation and infarct volume	-	[117]
	Glioblastoma/Temozolomide-resistant U87MG and U251 cells	Improved sensitivity of resistant cells to temozolomide	Inhibited upregulation of NEDD4-1- (induces PTEN, suppresses Akt/Nrf2/HO-1)	[118]

* Stroke denotes only ischemic stroke.

## Data Availability

Not applicable.
